# Pathological phimosis is associated with foreskin immune cell infiltration but not microbiota composition

**DOI:** 10.1128/msphere.00725-25

**Published:** 2026-03-25

**Authors:** Rachel Penney, Lane B. Buchanan, Jorge Rojas-Vargas, Jeff Lin, Yazan Khan, Jacob Davidson, Claire A. Wilson, Vera Tai, Thatiane A. Russo, Thomas J. Hope, Hannah Wilcox, Bern Monari, Jacques Ravel, Kait F. Al, Sumit Dave, Peter Zhan Tao Wang, Jeremy P. Burton, Jessica L. Prodger

**Affiliations:** 1 Department of Microbiology and Immunology, Schulich School of Medicine and Dentistry, Western University 6221, London, Ontario, Canada; 2 Centre for Human Microbiome and Probiotic Research, Lawson Research Institute 151158 , London, Ontario, Canada; 3 Department of Biology, Western University 6221, London, Ontario, Canada; 4 Division of Pediatric Surgery, Children’s Hospital, London Health Sciences Centre 10033 , London, Ontario, Canada; 5 Feinberg School of Medicine, Cell and Developmental Biology Department, Northwestern University 12244, Chicago, Illinois, USA; 6 Department of Microbiology and Immunology, Institute for Genome Sciences, University of Maryland, School of Medicine 1068 , Baltimore, Maryland, USA; 7 Division of Urology, Department of Surgery, Western University 6221, London, Ontario, Canada; 8 Department of Epidemiology and Biostatistics, Schulich School of Medicine and Dentistry, Western University 6221, London, Ontario, Canada; University of Michigan, Ann Arbor, Michigan, USA

**Keywords:** pediatric immunology, circumcision, microbiome, phimosis

## Abstract

The penile microbiota has been implicated in genital inflammation and increased risk of HIV, HPV, HSV-2, and female-partner bacterial vaginosis in adult males, yet its development during childhood and potential role in pediatric foreskin pathologies remain unknown. We characterized the coronal sulcus microbiota of 75 pediatric males (median age 8.5 years; 43% with pathological phimosis) before and after circumcision and compared these profiles to 56 uncircumcised adult men. Pediatric penile microbiota were highly diverse, dominated by strict and facultative anaerobes, and loosely structured compared to adults, who exhibited two distinct, ecologically organized communities. Circumcision markedly reduced anaerobic taxa and increased *Corynebacterium* and *Staphylococcus*. Pathological phimosis (the inability to retract the foreskin due to scarring) was associated with increased densities of CD3+ T cells, CD4+ cells, and CD11c+ dendritic cells, suggesting an adaptive immune mechanism; however, pathological phimosis was not associated with microbiota composition. Nonetheless, *Mobiluncus* was negatively correlated with CD11c+ dendritic cells, while *Campylobacter* and *Peptoniphilus* were negatively correlated with CD56+ NK cells, suggesting microbe-immune interactions. Our data suggest pathologic phimosis is driven by adaptive immune responses but not by specific bacteria; the pathology may be driven by differences in host responses to bacteria or by other stimuli, such as fungal antigens. Our data also demonstrate that the transition to adulthood is accompanied by reorganization of penile communities into structured types previously linked to infection risk, highlighting puberty as a potential window for interventions that promote protective adult microbiota and improve lifelong sexual and reproductive health.

**IMPORTANCE:**

The human penis hosts complex bacterial communities that can influence inflammation, infection risk, and sexual health, but little is known about how these communities form early in life or whether they contribute to childhood foreskin inflammatory disorders. We combined 16S rRNA sequencing with quantitative microscopy to investigate the penile microbiota in boys and its relationship to pathological phimosis, a common condition marked by foreskin scarring. We found that phimosis is associated with infiltration of T cells and dendritic cells, indicating an adaptive immune process, but with no associations with specific bacteria. We also show that penile microbiota reorganize during puberty into structured community types previously linked to HIV and sexually transmitted infection risk. These findings suggest that childhood pathologic phimosis is mediated by adaptive immune responses rather than driven by specific bacterial communities and identify puberty as a critical period for shaping adult penile microbiota, with implications for lifelong genital health.

## INTRODUCTION

The uncircumcised penile microbiota in adults is comprised of a diverse community of predominantly anaerobic bacteria (1, 2). Male circumcision drastically alters the penile microbiota and is protective against urinary tract infections, penile cancer, and viral sexually transmitted infections (STIs) (2–9). In adults, the abundance of certain anaerobic bacteria in the sub-preputial microbiota correlates positively with markers of immune activation and the density of immune cells in foreskin tissue (10). Specifically, six species belonging to *Prevotella, Peptostreptococcus,* and *Dialister* have been associated with increased IL-8 in the sub-preputial space, increased density of T cells in the inner foreskin, decreased expression of epithelial junction proteins (11), and increased risk of acquiring HIV (10). The abundance of these anaerobes has also been associated with increased epithelial thickness and proliferation, which is a characteristic of inflammatory skin conditions like atopic dermatitis (11, 12). Adult circumcision significantly reduces penile anaerobes and increases bacteria more typical of the skin, including *Corynebacterium*, and decreases penile inflammation (2, 13). However, there are limited data on the penile microbiota of prepubescent males (14, 15), the effect of the microbiota on host immune responses, or how circumcision alters this relationship.

Pathological phimosis (the inability to retract the foreskin due to inflammation or scarring) is observed in 0.6% of pediatric males before 15 years of age (16, 17). Pathological phimosis usually presents as a tightened, white, fibrous ring (cicatrix) surrounding the opening of the foreskin and is associated with pain, bleeding, recurrent urinary tract infections, and inflammation (16). Some studies show that topical corticosteroids can help with mild scarring (16). When steroids fail or the scarring is too severe, phimosis can be treated effectively by circumcision (16, 18). Despite the prevalence of pediatric phimosis and its impact on health outcomes, the etiologic agents of foreskin inflammation and scarring remain largely unknown. We hypothesized that the inflammation causing pathological phimosis might be driven by the composition of the penile microbiota.

To address these knowledge gaps, we characterized the penile microbiota and foreskin inflammation in pediatric participants with and without phimosis, examined the effect of circumcision on the microbiota, and compared pediatric penile microbiota composition to a previously characterized cohort of adult males.

## MATERIALS AND METHODS

### Pediatric participants and sample collection

Participants were males under 18 years of age undergoing penile circumcision at the Victoria Hospital in London, Ontario, between May 2019 and September 2022. Participants were classified as pathological phimosis (phimosis with scarring), non-elective circumcision (phimosis without scarring at an advanced pediatric age), or elective circumcision (no phimosis or other medical indication).

The study consisted of two visits, one at the time of male circumcision and an optional visit 6 weeks post-circumcision. Participant history was collected at each visit, including prior probiotic and antibiotic use. The survey and its results were housed on a secure Research Electronic Data Capture platform at London Health Sciences Centre (19, 20).

Penile swabs were collected from the coronal sulcus, the area at the base of the glans penis, into a BBL Vacutainer Anaerobic Specimen Collector (Becton Dickinson) and transported to the lab on dry ice for storage at −80°C until DNA extraction. For the swab collected at the time of surgery, the foreskin was retracted under anesthetic to access the coronal sulcus (prior to use of antiseptic). For eight participants, retraction of the foreskin was not possible even under anesthetic. For these participants, the swab was taken from the inner foreskin after a dorsal slit was made in the tissue. Following circumcision, foreskin tissues were transported to Western University. Visible blood vessels and clots were excised. Inner and outer foreskin tissue sections of 0.25 cm^2^ were placed into optimal cutting temperature (OCT) compound before freezing and storing at −80°C.

### Characterization of pediatric penile microbiota

The DNA from the pediatric penile swabs was extracted using Qiagen’s DNeasy 96 PowerSoil Pro Kit with modifications made to the protocol to optimize the extraction for low-abundance samples. These changes included centrifuging the plate for twice the time indicated on the manufacturer’s instructions and eluting the samples with 50 μL of Solution C6 (instead of 100 μL). DNA was stored at −20°C until PCR amplification. Negative controls were included in both the DNA extraction and PCR amplification processes. PCR amplification was completed using the Earth Microbiome universal primers, 515F (5′-GTGYCAGCMGCCGCGGTAA-3′) and 806R (5′-GGACTACNVGGGTWTCTAAT-3′), which are specific for the V4 variable region of the 16S rRNA gene (Fisher Scientific, Mississauga, ON, Canada) (21). Two microliters of DNA template, followed by 20 µL of GoTaq hot-start colorless master mix (Promega, Madison, WI, USA), was added to a 96-well 0.2 mL PCR plate (Axygen, Union City, CA, USA) containing 20 μL of pre-mixed forward and reverse barcoded primers (both at working concentrations of 1.6 μM), such that each sample contained a unique barcode combination. The reaction was briefly mixed by pipetting, then sealed with a foil plate cover (Axygen) and centrifuged for 2 min at 2,250 × *g*. Amplification was performed in a Techne thermocycler (Minneapolis, MN, USA) with a lid temperature of 105°C and the following cycle conditions: an initial warm-up of 95°C for 4 min, followed by 30 cycles of 95°C for 1 min, 52°C for 1 min, and 72°C for 1 min, followed by a final extension step at 72°C for 5 min. Amplicons were stored at −20°C until further processing. Additional library preparation and sequencing was carried out at the London Regional Genomics Center (http://www.lrgc.ca). Amplicons were quantified using PicoGreen (Invitrogen, Fisher Scientific) and pooled at equimolar concentrations before cleanup using QIAquick (Qiagen). Using the 600-cycle MiSeq v.3 Reagent Kit (Illumina, San Diego, CA, USA), 2 × 260 bp paired-end sequencing was performed with the addition of 7.5% ɸX-174 at a cluster density of ~1,100. Sequence reads were exported as fastq files, demultiplexed using cutadapt (v.4.3) (22), quality filtered, trimmed, and denoised to infer exact amplicon sequence variants (ASVs) with DADA2 (v.1.26.0) (23) in R (v.4.2.1, R Foundation for Statistical Computing, Vienna, Austria). Paired-end reads were merged, chimeras were removed, and taxonomy was assigned using the SILVA database (v.138.2) (24). Potential contaminant ASVs were identified with the R package decontam (v.1.24.0), whereby the DNA extraction and PCR negative controls, as well as sample DNA concentrations, were utilized in the prevalence and frequency modes, respectively. ASVs identified as contaminants by both modes were removed, as were ASVs with assigned taxonomy to the kingdom “Eukaryota,” the order “Chloroplasts,” or the family “Mitochondria.” The ASV table was further pruned of samples containing less than 1,000 reads and ASVs with a proportional abundance less than 1% across all samples.

### Characterization of adult penile microbiota

Pediatric penile microbiota data were compared to previously published microbiota data from a cohort of 56 adult males (>18 years of age) (25). In brief, coronal sulcus swabs were self-collected by uncircumcised males attending a urology clinic at St. Joseph’s Hospital in London, Ontario. Canada. Swabs were placed in 1 mL of Qiagen C2.1 solution, a proven nucleic acid preservative that maintains the stability of both DNA and RNA at room temperature for up to 1 month. DNA extraction was performed using a MagAttract PowerMicrobiome DNA/RNA kit (Qiagen) on a Microlab STAR (Hamilton). The V3-V4 regions of the 16S rRNA gene were amplified utilizing a two-step PCR workflow with dual-index barcoding, targeting the V3-V4 hypervariable region using IGS-standard primers (319F/806R) with Illumina sequencing adapters and heterogeneity spacers, as previously described (26). Negative controls, including extraction blanks and no-template PCR controls, were processed in parallel with study samples. Amplicons were pooled at equimolar concentrations, purified, and sequenced on an Illumina NextSeq 1000, generating 300 bp paired-end reads. Sequence data were demultiplexed and the primer sequences removed. As with the pediatric samples, reads were quality filtered, trimmed, and ASVs were inferred using the DADA2 pipeline (23), which includes model-based error correction and removal of chimeric sequences. Error models were learned from the data within each sequencing run; no additional correction for Illumina quality-score binning was required for these samples. Taxonomy was assigned using the SILVA 16S rRNA gene reference database (v.138.2) (24).

### Foreskin tissue processing and quantitative immunofluorescent microscopy

Foreskin tissues in OCT were sectioned (8 µm–10 µm) and fixed for 10 min in 3.7% formaldehyde in 0.1 M PIPES buffer, pH 6.8. Sections were blocked with 10% normal donkey serum (Avantor), 0.1% Triton X-100 (Sigma-Aldrich), and 0.1% sodium azide (Sigma-Aldrich) in PBS. Antibodies used are listed in Table S1. Tissue sections were incubated with primary antibodies at 37°C for 1 h and with secondary antibodies at 25°C for 30 min. Sections were washed with PBS between incubations. Fluoromount-G Mounting Medium with DAPI (Thermo Fisher) was used to mount coverslips and visualize cell nuclei. Tiled images of whole foreskin tissue sections were imaged (200×) using a DM5500B fluorescence microscope (Leica) and exported as TIF files. The excitation and emission filters are listed in Table S2. Image quantification methods have been described in detail previously (11, 27, 28).

The epidermis was manually segmented, and folds and artifacts excluded, using full tissue section images in ImageJ software (v.2.1.0). A custom workflow was designed to acquire the following regions using the epidermal tracings: surface of the tissue, basal edge of the epidermis, and a region of the dermis extending 100 µm from the epidermis. Cells expressing the surface markers CD3 and CD4 were quantified using a previously published machine learning cell segmentation workflow (28), which uses the ImageJ STARDIST plugin (29). The analysis was performed on a Windows 10 and Ubuntu 22.04 dual-boot configured workstation equipped with an NVIDIA GeForce RTX 3090 GPU. This methodology was adapted to detect new cell surface proteins CD56, CD68, CD11c, and CD207. A supplementary manual correction workflow was then implemented with the CellCounter tool in ImageJ (v.2.1.0). Surface areas of the entire epidermis and the 100 µm region of dermis were measured to calculate cells per mm^2^ of tissue (density). Following the same fixation protocol described above, mast cells were identified via immunohistochemistry performed using a Bond-Max Automated Staining System (Leica). Primary antibody incubation was carried out for 15 min using either a 1:200 dilution of anti-rabbit CD117 (c-Kit) antibody (Agilent Technologies) or a 1:1,000 dilution of anti-mouse tryptase antibody (BioLegend). This was followed by a 16 min post-primary polymer incubation and a 5 min peroxidase block. 3,3′-Diaminobenzidine (DAB) was incubated for 10 min. Slides were counterstained with hematoxylin for 5 min, then dehydrated and coverslipped. Imaging was performed at 20× (0.46 µm/pixel) using a Hamamatsu NanoZoomer 2.0-HT digital slide scanner (Hamamatsu Photonics, Japan). Whole tissue scans were analyzed in QuPath software (v.0.3.2). Positive cell expression of CD117 and tryptase was manually marked throughout the entire tissue using the Point tool. Whole tissue areas were outlined using the Brush tool to calculate cells per mm² of tissue (density). All image processing and analyses were performed by an investigator blinded to participant group and microbiota data.

### Statistical analyses

All statistical analyses were performed in R (v.4.2.1) with tidyverse (v.2.0.0) (30) for data handling and ggplot2 (v.3.5.0) (31) for graphics. Microbiome-specific tasks used phyloseq (v.1.42.0) (32) to import and filter count tables, as well as calculate Shannon diversity and Pielou evenness. Core genera (>1% relative abundance in ≥50% of samples) were calculated with the microbiome (v.1.20.0) package. The vegan package (v.2.6.4) was used to compute Aitchison distances and test group separation with envfit (v.1.0.1) for correlation heat maps. ALDEx2 (v.1.30.0) (33) and MaAsLin2 (v.1.18.0) (34) were used for compositional and correlation testing; Hmisc (v.4.9-1) was used for classical Spearman correlation coefficients; and igraph (v.1.5.1) was used to derive cohesion and connectedness metrics from those correlations, and to relate CLR-transformed genus abundances to immune-cell densities in foreskin tissue.

Samples from the pediatric cohort were sequenced using an Illumina MiSeq, while adult samples were sequenced using an Illumina NextSeq. The mean sequencing depth of the uncircumcised pediatric samples was 47,743 reads, whereas it was 190,591 reads for the adults. Therefore, we did not directly statistically compare abundances of bacteria between the two cohorts. For genus-genus association structures within each cohort, the 30 most abundant genera in each cohort were correlated pairwise; Spearman correlations were FDR-adjusted and visualized with a heatmap. For the 22 genera common to both top 30 lists, cohort-specific shifts in association strength were quantified as Δρ = ρ_adult – ρ_pediatric and the distributions of |ρ| and Δρ compared with Wilcoxon rank-sum tests. Sample-level positive and negative cohesion and genus-level connectedness were calculated from these matrices and compared between cohorts with Wilcoxon rank-sum tests.

Age differences among groups (pathological phimosis, non-elective circumcision, elective circumcision) were examined with Kruskal-Wallis tests and ethnicity distributions with Fisher’s exact test (α = 0.05). Microbiota composition was explored by principal-component analysis of CLR-transformed Aitchison distances; Shannon diversity and Pielou evenness were compared by indication for circumcision and pre- vs post-circumcision using Student’s *t*-tests, after verifying normality by the Shapiro-Wilk test and QQ-plots. Genera were deemed differentially abundant between pre- and post-circumcision samples when ALDEx2 effect size exceeded |0.5| and FDR-corrected Wilcoxon *P* ≤ 0.001.

After retaining genera detected in ≥30% of samples, associations between bacterial genus and immune cells were evaluated with two complementary, cross-validating approaches: (i) Generalized additive model GAM pipeline: raw counts (rows = samples, columns = genera) were zero-replaced with the CZM replacement and CLR-transformed (compositions::clr). Each genus was then modeled against an immune-cell density with a separate GAM, CLR ~ s(marker, k = 3, bs = “cs”), fitted in mgcv by restricted maximum likelihood with automatic smooth-term selection (select = TRUE). Thin-plate cubic splines allowed gentle curvature while penalizing over-fitting; diagnostics (gam.check, k.check) confirmed adequate basis size for all significant fits (k-index ≈ 1, *P* > 0.15). (ii) ALDEx2 pipeline: the same count table, transposed so that columns represented samples, was transformed to aldex.clr (v.1.30.0). For each immune-cell marker, aldex.corr computed Spearman correlations between CLR values and marker densities. Only significant associations that coincided in both pipelines (FDR-adjusted *P* ≤ 0.05) were reported. The ALDEx2 CLR transformations were plotted as the mean values of the 128 replicates.

Densities of immune cells were compared between (i) epidermis and dermis, with paired Wilcoxon tests, and (ii) between elective and pathological phimosis groups, with unpaired Wilcoxon rank-sum tests. Unless otherwise specified, *P*-values were adjusted for multiple testing with the FDR method, and significance was set at *P* < 0.05.

## RESULTS

### The penile microbiota of uncircumcised pediatric males

Demographics of the 75 pediatric participants are presented in Table 1. The median age of participants was 8.5 years old, with a range of 7 months to 17.3 years. Participants predominantly self-identified as Caucasian (47.8%) or Middle Eastern (30.4%). Thirty-one percent of participants were able to retract their foreskin and were undergoing circumcision for elective reasons. Two participants had used topical steroids in the past 30 days (2.7%). No participants had used antibiotics within four weeks of baseline swab collection and circumcision. There were no significant differences in age or ethnoracial identity by indication for circumcision (all *P* > 0.1).

**TABLE 1:** Participant demographics

Characteristics	Participants (*n* = 75)
Age	
0–3	12 (16.0%)
4–8	20 (26.7%)
9–12	23 (30.7%)
13–18	20 (26.7%)
Ethnoracial identity	
Caucasian	56 (74.7%)
Middle Eastern	8 (10.7%)
Asian	7 (9.3%)
African American	2 (2.7%)
Other	2 (2.7%)
Indication for circumcision	
Elective	23 (30.7%)
Non-elective	20 (26.7%)
Pathological phimosis	32 (42.7%)

**Fig 1:**
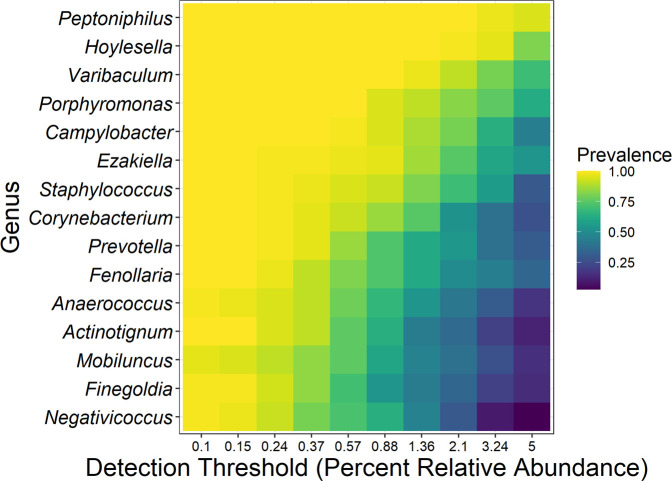
Core bacteria of the penile microbiota of uncircumcised pediatric males. The core microbiota (genera present at >1% proportional abundance in at least 50% of samples) of the pediatric (<18 years; *n* = 75) coronal sulcus consisted of 15 genera. Coloring denotes the proportion of individuals who had a relative abundance at or above the stated threshold on the *x*-axis. The core microbiota was determined using the core members function in the microbiome package in R.

The core microbiota—defined as genera of bacteria detected at greater than 1% proportional abundance in at least 50% of samples—was found to be comprised of 15 genera (Fig. 1). The five genera with the highest median proportional abundance were all obligate anaerobes: *Peptoniphilus* (12.55%), *Hoylesella* (11.75%), *Varibaculum* (7.09%), *Ezakiella* (6.24%), and *Porphyromonas* (5.84%) (Table S3).

### Comparison of the uncircumcised penile microbiota of pediatric vs adult males

**Fig 2:**
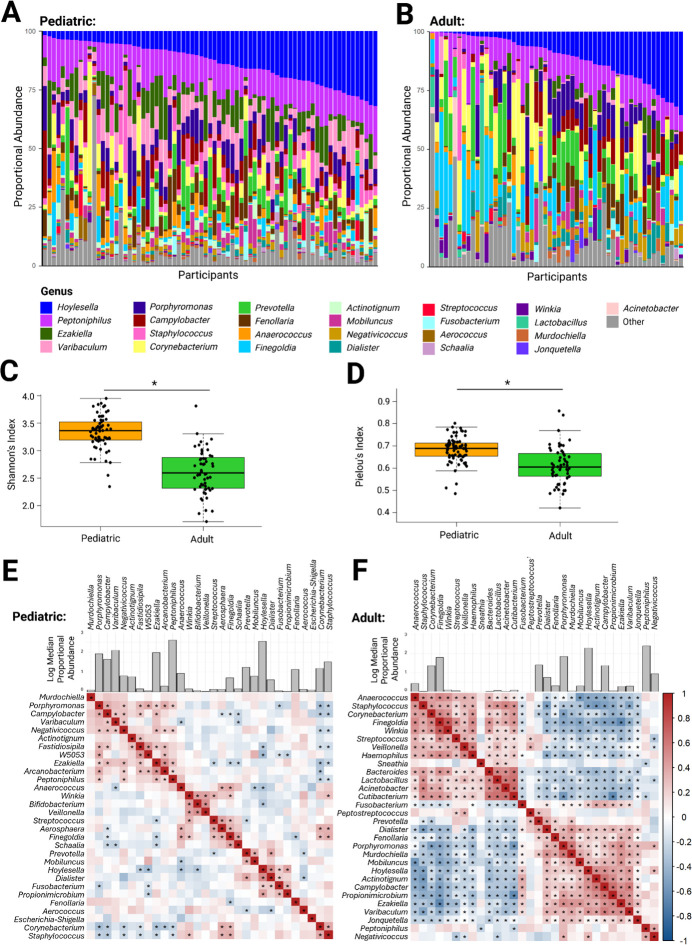
Pediatric sub-preputial microbiota are less structured, more diverse, and more even than adult penile microbiota. Stacked bar plots of the proportional abundances of the (**A**) pediatric (*n* = 75) and (**B**) adult (*n* = 56) uncircumcised penile microbiota are displayed, where each bar corresponds to one participant. The pediatric penile microbiota is significantly more diverse (**C**, Shannon’s index) and even (**D**, Pielou’s index) than adults (* indicates *P* < 0.05 by Student’s *t*-test). A matrix of the Spearman correlations of the top 30 most abundant genera in the (**E**) pediatric and (**F**) adult microbiota accompanied by a histogram of the logarithmic median proportional abundances of each genus (* indicates FDR-adjusted *P* < 0.05).

To identify unique characteristics of the pediatric microbiota, we compared the microbiota of pediatric participants to a previously published cohort of adults living in the same region of Ontario, Canada (25). Many of the same genera were present in both the pediatric and adult penile microbiota, but in different proportional abundances (Fig. 2A and B). Median relative abundance of *Varibaculum, Ezakiella,* and *Staphylococcus* was higher in the pediatric cohort (Δ = 6.76%, 5.93%, and 3.52%, respectively), whereas the relative abundance of *Finegoldia* was higher in the adult cohort (Δ = 4.06%) (Table S3). We next assessed community evenness, the degree to which taxa are evenly distributed within samples, and diversity, the overall variety of taxa present in the community. Pediatric microbiota exhibited both significantly higher evenness (median Pielou’s index: 0.689 vs 0.605; *P* = 6.2 × 10^−8^) and diversity (median Shannon’s index: 3.36 vs 2.59; *P* = 5.2 × 10^−16^) (Fig. 2C and D) than adult microbiota; however, within pediatric samples, we found no associations between age and evenness or diversity (both *P* > 0.3).

To assess differences in microbial community structure between pediatric and adult penile microbiomes, we generated correlation matrices of the top 30 genera with the greatest mean relative abundances in the pediatric and adult cohorts (22 genera were shared), and clustered genera based on correlations (Fig. 2E and F). Spearman correlation ρ and FDR-adjusted *P*-values are presented in Table S4.1 through 4.4. In adults, two well-defined clusters emerged: one dominated by *Finegoldia*, *Corynebacterium*, and *Anaerococcus* and another by *Prevotella*, *Hoylesella* (comprised of species previously classified as *Prevotella*), *Peptoniphilus*, *Porphyromonas*, and *Campylobacter*. The two clusters were largely anti-correlated, indicating distinct communities. Pediatric samples showed much weaker clustering, reflected by a lower median absolute correlation (|ρ| = 0.11 in children vs 0.45 in adults, *P* < 2 × 10⁻¹⁶).

To quantify these differences, we calculated community cohesion and connectedness, metrics that integrate all pairwise correlation values into sample-level (cohesion) and taxon-level (connectedness) scores. Cohesion captures the overall strength of positive and negative co-occurrence within each sample, while connectedness measures how strongly each genus relates to all others across the data set. Adult communities exhibited significantly higher positive and negative cohesion than pediatric communities (median positive cohesion = 0.152 vs 0.147, *P* = 0.04; mean negative cohesion = –0.134 vs –0.112, *P* = 1 × 10^−10^), and greater connectedness (median positive = 0.169 vs 0.143, *P* = 0.04; median negative = –0.148 vs –0.104, *P* = 0.001), indicating that adult penile microbiota form a more tightly organized network, with stronger cooperative guilds and sharper exclusions among genera, whereas pediatric communities remain comparatively diffuse and weakly interconnected.

To identify bacteria driving this pattern, for the 22 genera shared by both cohorts, we compared the Spearman correlation of every genus pair between adults and children (Δρ = ρ adult – ρ ped, Table S5). A positive Δρ indicates a correlation that became more positive (or less negative) in adults compared to pediatrics, while a negative Δρ denotes a correlation that became more negative (or less positive). The largest positive shifts were observed for *Propionimicrobium–Porphyromonas* (Δρ = +0.64) and *Mobiluncus–Ezakiella* (Δρ = +0.62), each moving from a weak negative association in children (ρ ≈ –0.2) to a moderate co-occurrence in adults (ρ ≈ 0.4–0.5). In contrast, *Staphylococcus–Hoylesella* and *Staphylococcus–Propionimicrobium* (both Δρ = –0.59), and *Mobiluncus–Finegoldia* (Δρ = –0.57), shifted from weakly positive in children to negative in adults. Shifts that strengthened an existing relationship without changing its sign were also common, most notably *Propionimicrobium–Campylobacter* (Δρ = +0.57), which reinforced a positive link, and *Dialister–Staphylococcus* (Δρ = –0.52), which intensified an already negative association.

### Effect of circumcision on the pediatric penile microbiota

A total of 51 pediatric participants provided an optional second swab at approximately 6 weeks post-circumcision; demographics of this subgroup are presented in Table 2. The median age of this subgroup was 8.6 years (range: 7 months–17.3 years), the majority identified as Caucasian (76.5%), and 23.5% were undergoing circumcision for elective reasons. Two participants had used topical steroids in the past 30 days (3.9%). There were no significant differences in age or ethnoracial identity by indication for circumcision (all *P* > 0.1).

**TABLE 2:** Demographics of participants with pre- and post-circumcision data

Characteristic	Participants (n = 51)
Age	
0–3	6 (11.8%)
4–8	14 (27.5%)
9 –12	16 (31.4%)
13–18	15 (29.4%)
Ethnoracial identity	
Caucasian	39 (76.5%)
Middle Eastern	6 (11.8%)
Asian	4 (7.8%)
African American	1 (2.0%)
Other	1 (2.0%)
Indication for circumcision	
Elective	12 (23.5%)
Non-elective	13 (25.5%)
Pathological phimosis	26 (51.0%)

**Fig 3:**
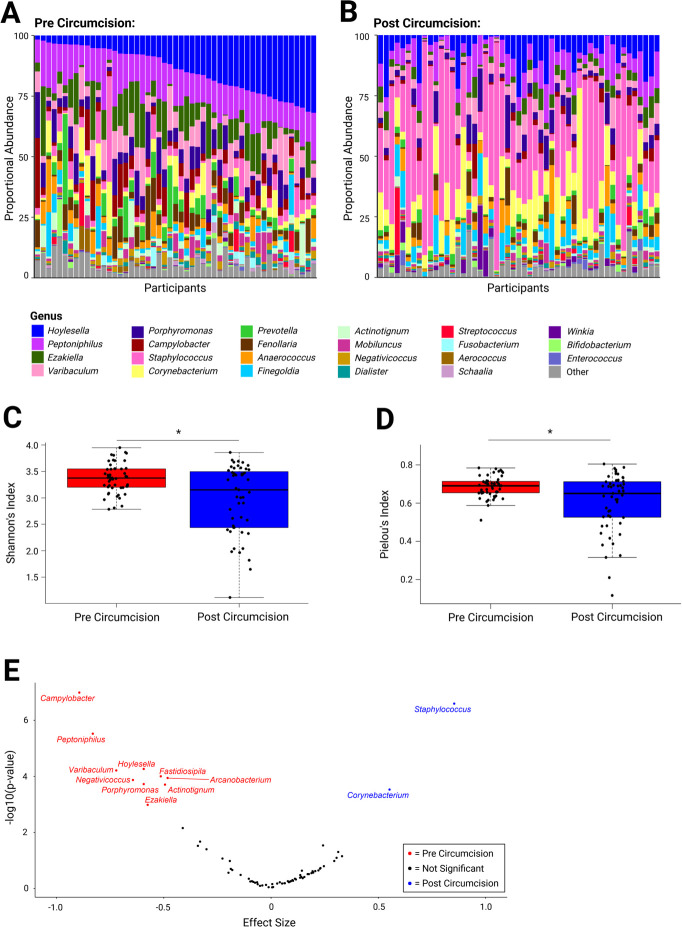
Overall diversity and anaerobic bacteria are decreased by pediatric penile circumcision. Stacked bar plots of the proportional abundance of the top 30 most abundant genera of penile bacteria pre-circumcision are displayed for pediatric participants pre- (**A**) and post-circumcision (**B**). Pre-circumcision, participants are arranged by the relative abundance of *Hoylesella* (the most abundant genus); participant order is maintained in the post-circumcision plot (*n* = 51). Shannon’s diversity (**C**) and Pielou’s evenness (**D**) decreased post-circumcision (Wilcoxon test). (**E**) Volcano plot illustrating differentially abundant genera pre- and post-circumcision. Each point represents a single genus; genera that were significantly enriched prior to circumcision are colored red, while genera enriched after circumcision are colored blue (effect size > |0.5| and Benjamini-Hochberg-corrected Wilcoxon rank-sum test *P*-value ≤0.001).

Stacked bar plots showing the relative abundances of the most abundant genera are displayed in Fig. 3. Circumcision significantly decreased bacterial diversity (median Shannon’s alpha index: 3.37 vs 3.15, *P* = 0.012, Fig. 3C) and community evenness (median Pielou’s index: 0.69 vs 0.65; *P* = 0.02, Fig. 3D). Circumcision resulted in significant decreases in the relative abundances of 10 genera: *Campylobacter*, *Peptoniphilus*, *Hoylesella*, *Varibaculum*, *Negativicoccus*, *Porphyromonas*, *Ezakiella*, *Fastidiosipila*, *Arcanobacterium,* and *Actinotignum*, and significant increases in the relative abundances of two genera: *Staphylococcus* and *Corynebacterium* (Fig. 3E and Table 3).

**TABLE 3:** Change in prevalence and proportional abundance of taxa after circumcision

Phylum	Genus	Effect size^*a*^	Adj. *P*-value^*b*^	Prevalence (%)^*c*^		Median proportional abundance (%)	
				Pre	Post	Pre	Post
Pseudomonadota	*Campylobacter*	−0.93	<0.0001	94	55	4.40	1.36
Bacillota	*Peptoniphilus*	−0.81	<0.0001	100	98	13.31	7.05
Actinomycetota	*Varibaculum*	−0.71	0.0001	100	92	7.09	3.28
Bacillota	*Negativicoccus*	−0.63	0.0001	61	22	1.21	0.00
Bacteroidota	*Porphyromonas*	−0.61	0.0002	94	88	5.84	2.94
Bacteroidota	*Hoylesella*	−0.59	0.0001	100	86	13.80	6.40
Bacillota	*Ezakiella*	−0.56	0.0010	92	80	7.09	3.04
Bacillota	*Fastidiosipila*	−0.52	0.0001	12	0	0.00	0.00
Actinomycetota	*Arcanobacterium*	−0.49	0.0001	16	0	0.00	0.00
Actinomycetota	*Actinotignum*	−0.49	0.0002	67	41	1.32	0.00
Actinomycetota	*Corynebacterium*	0.55	0.0003	84	98	2.66	7.80
Bacillota	*Staphylococcus*	0.87	<0.0001	88	96	3.52	21.75

^*a*^ Calculated using ALDEx2.

^*b*^ Benjamini-Hochberg-corrected *P*-values, calculated by Wilcoxon rank-sum test.

^*c*^ Genera were considered prevalent in individuals with >1% proportional abundance.

### Associations between microbiota and foreskin immune cells

**Fig 4:**
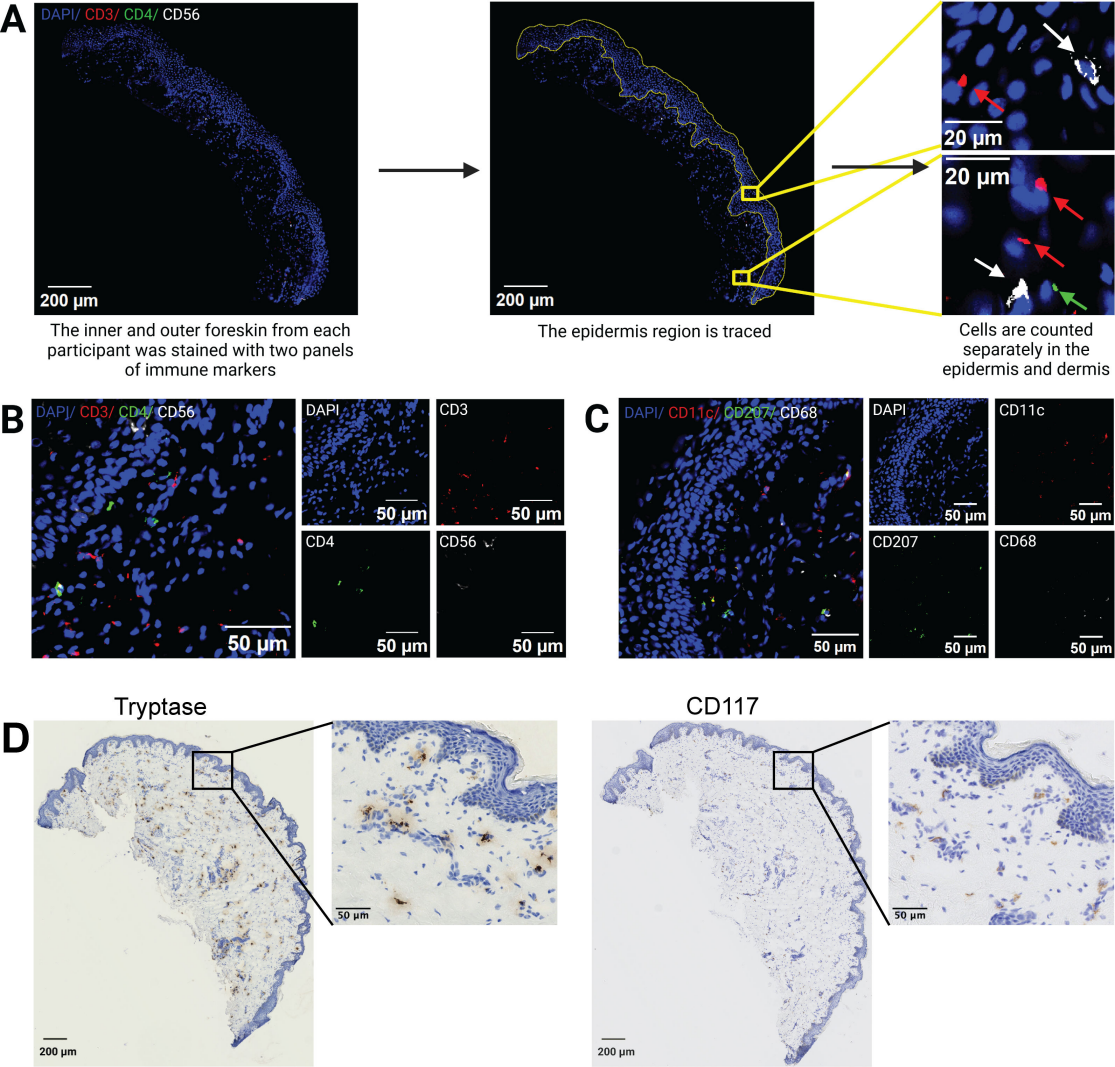
Representative microscopy images of immune cells in foreskin tissue. A schematic of immunofluorescence image processing is shown in panel **A**. Each foreskin was separated into inner and outer aspects and stained with two panels of cell markers. After the tissues were imaged, the epidermal and dermal regions (within 100 µm of the basement membrane) were manually segmented and analyzed separately. (**B**) Representative images of foreskin tissues stained for DAPI (blue), CD3 (red), CD4 (green), and CD56 (white); both composite and individual channels are displayed. (**C**) Representative images of foreskin tissues stained for DAPI (blue), CD11c (red), CD207 (green), and CD68 (white). (**D**) Representative images of foreskin tissues stained for CD117 or tryptase using DAB staining (brown) and counterstained with hematoxylin (blue).

We next assessed associations between pediatric penile microbiota and foreskin immune cells. Sections of the inner foreskin (IFS, skin adjacent to the glans and coronal sulcus when the foreskin is not retracted) and the outer foreskin (OFS, skin exposed to the air regardless of foreskin retraction) were sectioned, and immune cells were identified by microscopy (Fig. 4A). CD3 (T cells), CD56 (natural killer cells [NK]), and CD4 were identified in one panel (Fig. 4B); CD11c (inflammatory dendritic cells), CD207 (Langerhans cells), and CD68 (macrophages) were identified in a second panel (Fig. 4C), and mast cells were identified by Tryptase and CD117 histology (Fig. 4D). Cells were manually counted in the epidermis and dermis separately by an investigator blinded to tissue source.

In general, CD68+, CD3+, CD4+, and CD11c+ cells were significantly more abundant in the dermis compared to the epidermis (all adj *P* < 6 × 10^−5^, Fig. S1). CD68+ cells (putative macrophages) were the most abundant cell type in the dermis (median density IFS = 2,720 cells/mm^2^, OFS = 2,850 cells/mm^2^), followed by CD11c+ (putative dermal dendritic cells, median density IFS = 868 cells/mm^2^, OFS = 856 cells/mm^2^), CD3+ (T cells, median density IFS= 499 cells/mm^2^, OFS = 449 cells/mm^2^) and CD4+ (median density IFS= 442 cells/mm^2^, OFS= 472 cells/mm^2^) cells; CD56+ and CD207+ cells were rare in the dermis (IFS = 138 cells/mm^2^ and 98 cells/mm^2^, OFS = 119 cells/mm^2^ and 96 cells/mm^2^, respectively). CD207+ cells (Langerhans cells) were the most abundant cell type in the epidermis (median density IFS = 538 cells/mm^2^, OFS = 597 cells/mm^2^).

We next assessed correlations between immune cells and penile bacteria using two complementary analyses: GAM and ALDEx2. The two analytic approaches converged on three significant, non-linear, and negative associations between bacteria and inner foreskin immune cells (Fig. S2); no associations were observed between coronal sulcus bacteria and immune cells in the outer foreskin. The abundance of *Mobiluncus* correlated negatively with density of CD11c+ cells in the dermis (GAM edf = 1.40, adj *R*² = 0.20, adj *P* = 0.011; ALDEx2 ρ = –0.51, adj *P* = 0.002); while abundances of *Campylobacter* and *Peptoniphilus* correlated negatively with CD56+ cells in the epidermis (*Campylobacter*: GAM edf = 1.28, adj *R*² = 0.17, adj *P* = 0.041; ALDEx2 ρ = –0.47, *q* = 0.020. *Peptoniphilus*: GAM edf = 1.28, adj *R*² = 0.20, adj *P* = 0.025; ALDEx2 ρ = –0.46, *q* = 0.025). No significant associations were observed between bacteria and T cells (CD3), CD4+ cells, Langerhans cells (CD207), macrophages (CD68), or mast cells (CD117 or tryptase).

### Associations of pathological phimosis with foreskin microbiota and immune cells

To explore if sub-preputial bacteria contribute to pathological phimosis, we compared microbiota between participants who were undergoing circumcision to treat phimosis due to clinically apparent scarring (*n* = 32) and those who were undergoing elective circumcision (non-medical reasons with no clinically apparent inflammation or foreskin irritation; *n* = 23). Demographics of this subgroup of participants (*n* = 55) are presented in Table 4. There was no significant difference in age between participant groups (*P* = 0.34). Participants being circumcised for pathological phimosis were more likely to identify as Caucasian than those undergoing elective circumcision (*P* = 0.01).

**TABLE 4:** Demographics of pediatric participants included in analysis examining associations with pathological phimosis

Characteristic	Elective (non-medical; *n* = 23)	Pathological phimosis (*n* = 32)	*P*-value^*a*^
Age			0.34
0–3	7 (30.4%)	2 (6.3%)	
3–8	5 (21.7%)	10 (31.3%)	
8–12	4 (17.4%)	13 (40.6%)	
12–18	7 (30.4%)	7 (21.9%)	
Ethnoracial identity			0.01
Caucasian	11 (47.8%)	27 (84.4%)	
Middle Eastern	7 (30.4%)	1 (3.1%)	
Asian	3 (13.0%)	2 (6.3%)	
African American	1 (4.3%)	1 (3.1%)	
Other	1 (4.3%)	1 (3.1%)	

^*a*^ Differences in age assessed by Kruskal-Wallis; differences in ethnicity by Fisher’s exact test.

**Fig 5:**
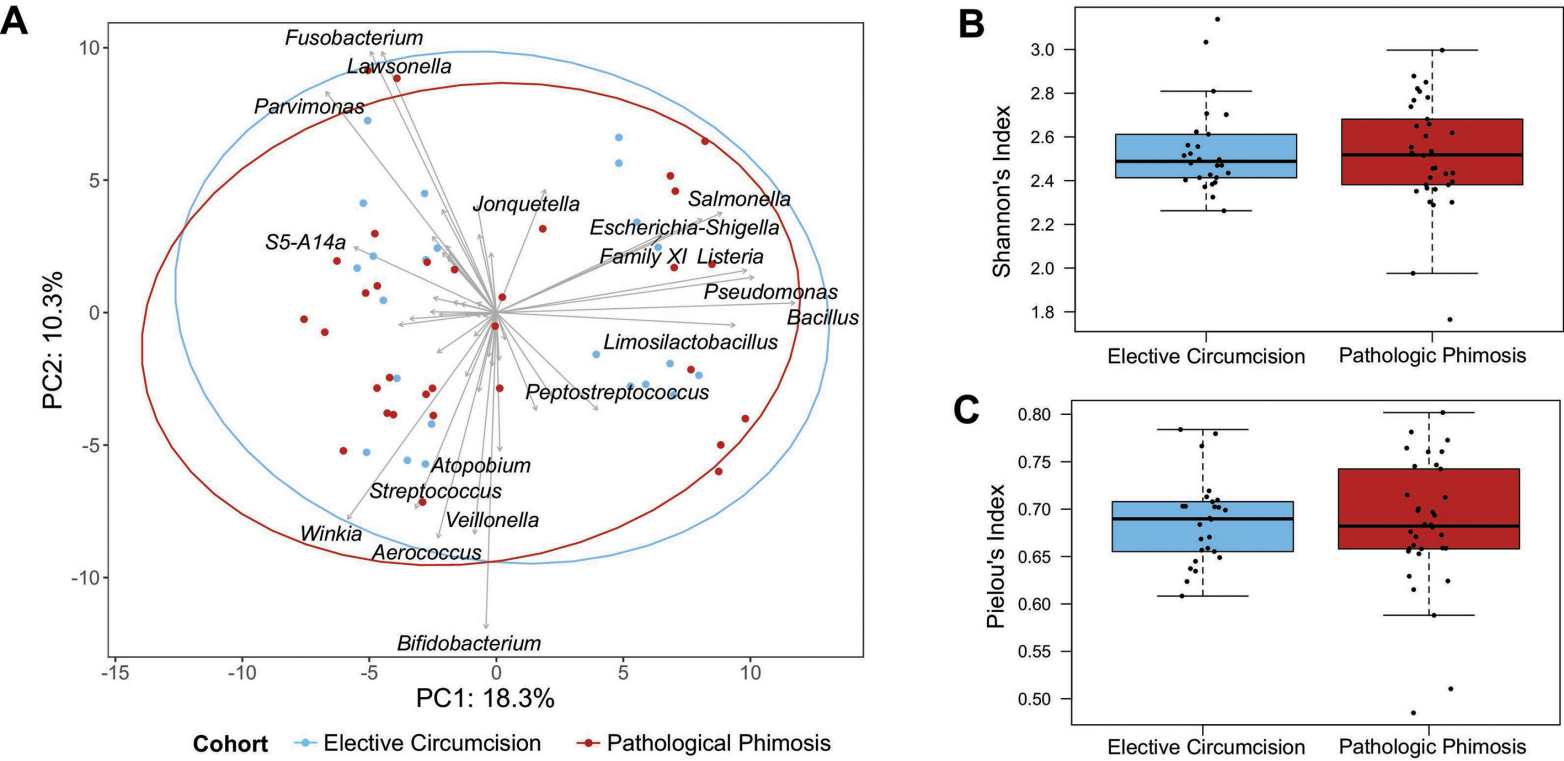
Pathological phimosis is not associated with pediatric penile microbiota. (**A**) Principal component analysis was performed on CLR-transformed Aitchison distances. Each data point indicates the composition of the pre-circumcision penile microbiota for one participant (*n* = 55). The distances between the samples represent the difference in microbial composition based on the first two principal components (PCs), which represent 28.6% of the total variance. The gray arrows depict the direction of association between each taxon and the two components, while arrow length depicts the relative strength of that association. Ellipses represent 95% confidence intervals around the group centroids. Pre-circumcision microbiota did not differ based on indication for circumcision (envfit *P* = 0.85). Pre-circumcision Shannon’s diversity (**B**) and Pielou’s evenness (**C**) also did not differ based on indication for circumcision (Wilcoxon test).

To assess differences in microbiota composition between participants with and without pathological phimosis, microbiota samples were CLR transformed, and Aitchison distances were visualized using a principal components analysis (PCA). No significant differences were observed in the composition of the microbiota between participant groups (envfit *P* = 0.85, Fig. 5). The group centroid for the elective cohort was located at PC1 = 0.333 and PC2 = 0.217, while the centroid for the pathological phimosis cohort was located at PC1 = −0.239 and PC2 = −0.156 in the PCA plot. These coordinates represent the mean positions of each group in the reduced dimensional space, summarizing overall group separation. Microbiota of participants with and without pathological phimosis were also similar based on diversity (median Shannon’s alpha index: 2.49 vs 2.52, *P* = 0.96; Fig. 5B) and community evenness (median Pielou’s index: 0.69 vs 0.68, *P* = 0.97; Fig. 5C).

**Fig 6:**
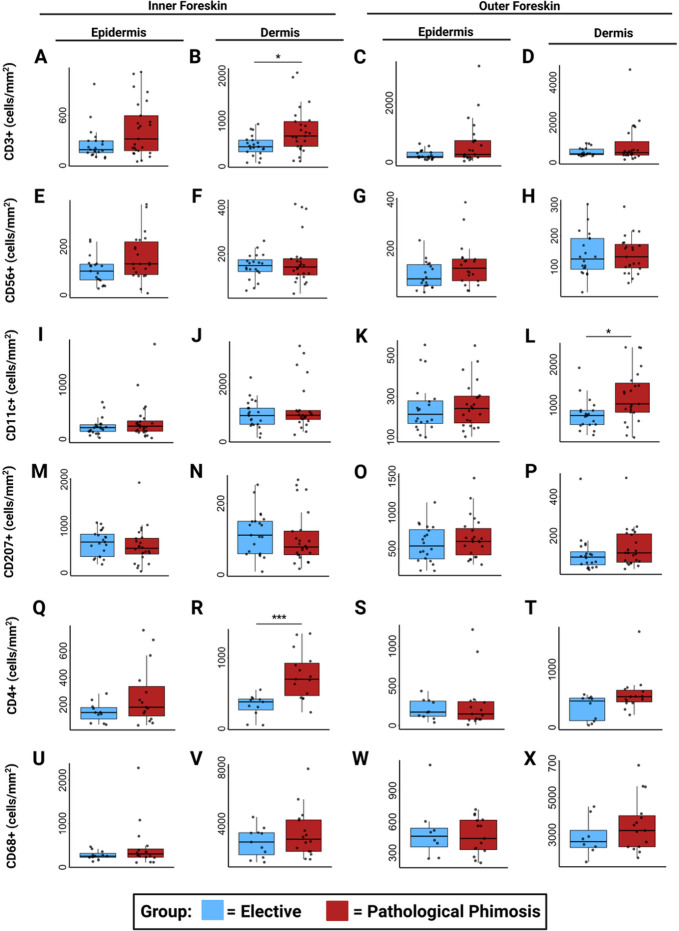
Increased densities of dermal CD3+, CD4+, and CD11c+ cells in the foreskins of participants with pathological phimosis. Cell densities were calculated using cell counts divided by tissue area (mm^2^) for whole tissue sections. Density of CD3+ (**A–D**), CD56+ (**E–H**), CD11c+ (**I–L**), CD207+ (**M–P**), CD4+ (**Q–T**), and CD68+ (**U–X**) cells was quantified in the epidermis and dermis of both the inner foreskin and outer aspects of the foreskin separately. Wilcoxon rank-sum tests were conducted to assess differences between participants with pathologic phimosis compared to participants undergoing elective circumcision (*0.01 < *P* < 0.05, ***0.001 < *P <* 0.0001)

**Fig 7:**
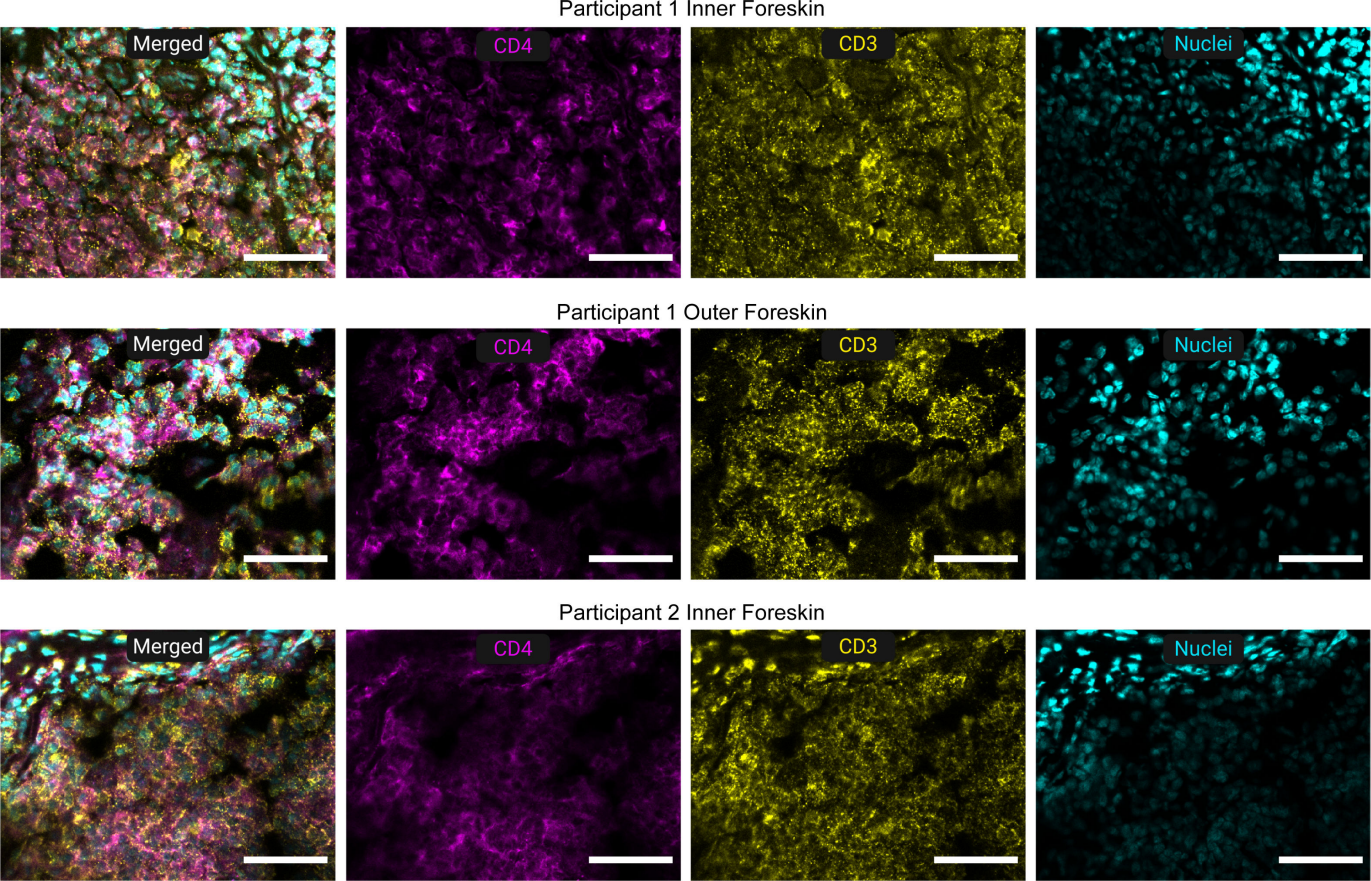
Representative images of CD4+ T cell clusters in participants with pathological phimosis. Inner and outer foreskin tissues were stained for CD3 (yellow), CD4 (magenta), and cell nuclei (cyan). Full tissue section imaging revealed large CD4+ T cell clusters in several participants, predominantly in the inner foreskin dermis and, less commonly, in the corresponding outer foreskin dermis. Scale bar: 50 µm.

Despite a lack of associations between pathological phimosis and bacteria, we did observe associations with immune cells (Fig. 6). In some participants with pathological phimosis, large clusters of immune cells, mainly CD4+ T cells, were observed in the inner and outer foreskin dermis (Fig. 7). Upon quantification, participants undergoing circumcision for pathological phimosis had significantly higher density of CD3+ (median density of 434 cells/mm^2^ for elective and 672 cells/mm^2^ for pathological phimosis) and CD4+ cells (median density of 378 cells/mm^2^ for elective and 697 cells/mm^2^ for pathological phimosis) in the inner foreskin dermis compared to participants undergoing elective circumcision (*P* = 0.016 and *P* = 0.00051, respectively). Additionally, participants with pathological phimosis had significantly higher median CD11c+ cell density of 1033 cells/mm^2^ in the dermis of the outer foreskin compared to elective participants with a median cell density of 747 cells/mm^2^ (*P* = 0.011). No significant associations were observed between indication for circumcision and foreskin density of putative macrophages (CD68), NK cells (CD56), Langerhans cells (CD207), or mast cells (CD117 or tryptase).

## DISCUSSION

The sub-preputial microbiota of uncircumcised pediatric males was highly diverse and dominated by strict and facultative anaerobes. However, it was loosely structured compared to the less diverse but more interconnected adult communities. Circumcision markedly altered this community, reducing many strict anaerobes and producing a microbiota dominated by *Corynebacterium* and *Staphylococcus*. Pathological phimosis was characterized by increased densities of T cells and dendritic cells in foreskin tissue but showed no associations with macrophages, Langerhans cells, NK cells, or mast cells. Thus, while pathological phimosis was clearly linked to immune cell infiltration, it was not associated with differences in the sub-preputial microbiota.

While the pediatric penile microbiota shared many taxa with adults, children exhibited significantly greater diversity and evenness. In youth, correlations between taxa were weak, whether positive or negative, indicating little ecological structuring. By contrast, adult microbiota formed two distinct communities: a gram-positive cluster (*Finegoldia*, *Corynebacterium*, and *Anaerococcus*) and a gram-negative cluster (*Prevotella*, *Hoylesella*, *Porphyromonas*, and *Campylobacter*). Genera within the same cluster were positively correlated, whereas those across clusters were generally negatively correlated. This pattern was absent in children, suggesting that penile microbial communities become more coordinated with age. The development of stronger microbial partnerships and antagonisms reflects ecological organization shaped by mutualism, competition, or niche filtering. In adults, *Prevotella*-dominated communities have been linked to inflammation, increased risk of viral STIs, and female-partner bacterial vaginosis, whereas *Corynebacterium*-rich communities are associated with lower risks (35). As these two community types appear to emerge during the transition to adulthood, this may provide an exciting opportunity for novel interventions aimed at promoting an optimal adult microbiota. Identifying the determinants of which community establishes may have important implications for adult sexual and reproductive health in both males and females.

We next examined how circumcision shapes the pediatric penile microbiota. Similar to previous reports in adults, we observed that circumcision reduced several strictly anaerobic genera and increased two aerobic/facultative anaerobic genera: *Corynebacterium* and *Staphylococcus* (2, 10). However, in contrast to previous reports in adults, we found higher relative abundance of *Staphylococcus* than *Corynebacterium* in pediatric males after circumcision, while the reverse has been observed in circumcised adults (*Corynebacterium* > *Staphylococcus*) (2, 10, 36). This is consistent with non-penile skin, where abundance of *Staphylococcus* has been reported to decrease with age (37).

Two previous studies have described the effect of circumcision on the coronal sulcus microbiota of healthy pediatric participants (14, 15). Mishra et al. characterized changes in the penile microbiota 6 weeks after circumcision in 11 toilet-trained pediatric participants from the United States (14), and Salazar et al. characterized the penile microbiota 1–2 weeks after circumcision in 30 neonates in Uganda (15). In agreement with the current study, both studies reported circumcision to be associated with reduced abundance of strict anaerobes and increased abundance of facultative anaerobes. However, Mishra et al. reported *Ralstonia* as the predominant genus post-circumcision (56%), in contrast to both our findings and those of Salazar et al., where *Ralstonia* was rare and *Corynebacterium* and *Staphylococcus* were the dominant genera. Interestingly, Salazar reported relatively high abundances of *Veillonella*, *Escherichia*, and *Bifidobacterium*, which we detected at very low abundance. Given that members of these genera are highly prevalent in the infant gut microbiota (38), it is possible that their presence among neonates—and absence in our relatively older cohort—may be due to diaper-wearing in the former.

Compared to males without phimosis (undergoing elective circumcision), pathological phimosis was associated with higher densities of foreskin CD3+, CD4+, and CD11c+ cells. CD3 marks T cells, CD4 is expressed on helper T cells and some antigen-presenting cells, and CD11c identifies dermal dendritic cells and inflammatory dermal and macrophage subsets (39). In contrast to T cells and dendritic cells, no differences were observed in cells expressing CD68, CD56, CD207, or CD117/tryptase between pathological and elective circumcision groups. These markers, expressed by macrophages, NK cells, Langerhans cells, and mast cells, are typically associated with antiviral defense or tissue remodeling. T cells have been previously reported to contribute to inflammation during lichen sclerosus, which can lead to scarring and pathological phimosis (40), and their enrichment along with inflammatory dermal dendritic cells suggests that pathological phimosis is driven by chronic antigenic stimulation. Although this aligns with our initial hypothesis that the penile microbiota could underlie the condition, we found no association between microbiota composition and pathological phimosis. In contrast, in cohorts focused on adults with lichen sclerosus-associated urethral stricture, significant differences in foreskin microbiota have been observed, including enrichment of *Acinetobacter*, *Pseudomonas,* and *Mycobacterium* and depletion of *Corynebacterium*, *Finegoldia*, and *Peptoniphilus* (41, 42). It is possible that the lack of association between pathological phimosis and microbiota in our study is due to the inclusion of participants with a less extreme clinical phenotype. In keeping with this, two studies examining men with lichen sclerosus, but not specifically urinary stricture, did not find robustly statistically significant associations (43, 44). An alternative (non-exclusive) explanation for the discrepancy with adult studies is that—in contrast to adults—our pediatric participants rarely had *Corynebacterium*/*Finegoldia* dominant communities, limiting our ability to detect an enrichment in these genera in healthy participants.

It is also possible that pathological phimosis is driven by bacterial antigens, but that the causative factor is differences in host responses to bacteria rather than microbiota composition. Studies examining host genetic factors (e.g., HLA type), T cell receptor repertoire, and antigen-specific responses of T cells will be important to determine whether phimosis is driven by host-microbe interactions. An alternative possibility is that the increased adaptive immune cells in pathological phimosis could target non-bacterial antigens, such as fungi, which have been implicated in other chronic inflammatory skin conditions. Future work should therefore also assess the penile mycobiome and host immune responses to fungal antigens.

Although we found no association between microbiota composition and pathological phimosis, our data revealed specific bacterial taxa that correlated with foreskin immune cell densities, consistent with adult studies demonstrating the capacity of the penile microbiota to shape local immunity (10, 11, 45, 46). *Mobiluncus* abundance was negatively correlated with dermal CD11c+ dendritic cells, while *Campylobacter* and *Peptoniphilus* were negatively correlated with epidermal CD56+ NK cells. This aligns with previous data from adults, where *Corynebacterium* abundance was associated with significantly lower expression of genes involved in neutrophil chemotaxis, supporting the idea that specific taxa can dampen pro-inflammatory immune pathways (47). These data suggest that, even in children, microbial composition influences the immune milieu of the foreskin. Although not linked to pathological phimosis in this cohort, such microbe-immune interactions may still influence susceptibility to viral STIs and the potential immunomodulatory effects of *Mobiluncus*, *Peptoniphilus*, and *Campylobacter* warrant further investigation.

This study has several limitations. Our definition of pathological vs non-pathological phimosis is based on the presence of visible scarring, which presents on a spectrum. As a result, participants classified as non-pathological phimosis may have had mild scarring that was not clinically apparent, and the degree of scarring within pathological phimosis varied. While our data revealed clear differences in microbial diversity and composition between pediatric and adult penile microbiota, the two cohorts were analyzed using different sequencing platforms (MiSeq vs NextSeq). However, our data show a lower diversity in adults, despite deeper sequencing, suggesting the observed patterns are biologically valid. Future longitudinal studies following participants through physical and sexual maturation are warranted to validate our cross-cohort observations and explore determinants to adult microbiota community type. Finally, our study was limited to single-marker identification of foreskin immune cells due to technical constraints, allowing relative quantification of major subsets but not functional phenotypes or full diversity. Future use of multiparameter flow cytometry, spatial transcriptomics, or single-cell sequencing could provide a more detailed view of immune responses in pathological phimosis.

This study characterized the pediatric penile microbiota and its relationship to foreskin immune cell composition and pathological phimosis. We found that while the pediatric microbiota is distinct from adults and significantly altered by circumcision, it was not associated with foreskin inflammation or scarring. Pathological phimosis was marked by increased densities of T cells and dendritic cells, pointing to an immune-mediated process that may be independent of bacterial composition or may be dictated by differences in host responses to bacteria. These findings underscore the importance of host immune responses in the pathogenesis of pathological phimosis. Importantly, our data also indicate that penile microbial communities are loosely organized in childhood but become structured into distinct adult community types around puberty. Because adult microbiota composition has been associated with risk of HIV, HPV, HSV-2, and female-partner bacterial vaginosis, puberty and sexual debut may represent a critical window for interventions that promote the establishment of protective, gram-positive–dominated communities. Future work should explore how host, microbial, and environmental factors interact during this transition, with the goal of informing strategies to improve lifelong sexual and reproductive health.

## Data Availability

All data tables have been deposited in GitHub and are publicly available at https://github.com/prodgerlab/TransBiota/tree/main/Neovaginal_microbiota_paper and https://github.com/prodgerlab/Pediatric_microbiota_paper. Any additional information required to reanalyze the data reported in this paper is available from the corresponding author, upon request.

## SUPPLEMENTAL MATERIAL

Supplemental Material msphere.00725-25-s0001.pdf Tables S1-S4 and Figures S1 and S2.

ASM does not own the copyrights to Supplemental Material that may be linked to, or accessed through, an article. The authors have granted ASM a non-exclusive, world-wide license to publish the Supplemental Material files. Please contact the corresponding author directly for reuse.
