# Supplementary material for: Pathological phimosis is associated with foreskin immune cell infiltration but not microbiota composition
Source: mSphere. 2026 Mar 25;11(4):e00725-25. doi: 10.1128/msphere.00725-25 (PMC13123712; doi:10.1128/msphere.00725-25)
Supplement: Supplemental Material — Tables S1-S4 and Figures S1 and S2. [file msphere.00725-25-s0001.pdf]

Supplementary Material

Supplemental Table 1. Antibodies used for immunofluorescence.

Target	1°/2°	Clone	Supplier	Host Species	Dilution	Fluorophore
CD3	1°	SP7	Abcam	Rabbit	Neat	None
CD4	1°	Polyclonal	R&D	Goat	1:10	None
CD56	1°	sc-7326	Santa Cruz	Mouse	1:100	None
CD207	1°	Polyclonal	R&D	Goat	1:20	None
CD11c	1°	EP1347Y	Abcam	Rabbit	1:250	None
CD68	1°	C68/684	Abcam	Mouse	1:500	None
Mouse IgG*	2°	Polyclonal	Fisher	Donkey	1:400	AF 488
Rabbit IgG*	2°	Polyclonal	Fisher	Donkey	1:400	AF 647
Goat IgG*	2°	Polyclonal	Fisher	Donkey	1:400	AF 568

*Binds both heavy and light IgG chains (H + L)

Supplemental Table 2. Excitation and emission filters for immunofluorescence microscopy.

Target	Leica Filter	Absorbance λ	Excitation Filter[a]	Emission λ	Emission Filter[a]
DAPI	CFP	358	436/20	461	480/40
AF 488	GFP	494	425/60	517	480 LP
AF 546	DSR	556	545/30	573	620/60
AF 647	Y5	650	620/60	665	700/75

LP long pass filter

λ wavelength, in nanometers

[a]Peak wavelength of light that passes through the filter and filter bandwidth, in nanometers

Supplemental Table 3. Proportional abundances of the 25 most bacterial genera in the Pediatric and Adult cohorts. Pediatric (n=75) and adult (n=56) uncircumcised penile microbiotas were quantified by 16S rRNA gene sequencing.

Phylum	Genus	Prevalence (%)		Median Relative Abundance (%)			
		Pediatric	Adult	Pediatric	Adult	Δ P-A	adj. p*
Bacillota	*Peptoniphilus*	100	100	12.55	10.18	2.37	**0.0016**
Bacteroidota	*Hoylesella*	100	98.2	11.75	8.99	2.76	0.0636
Actinomycetota	*Varibaculum*	100	82.1	7.09	0.33	6.76	**<0.0001**
Bacillota	*Ezakiella*	100	78.6	6.24	0.31	5.93	**<0.0001**
Bacteroidota	*Porphyromonas*	100	89.3	5.84	5.39	0.45	0.258
Pseudomonadota	*Campylobacter*	100	89.3	4.15	2.95	1.20	0.131
Bacillota	*Staphylococcus*	100	69.6	3.53	0.01	3.52	**<0.0001**
Bacteroidota	*Prevotella*	100	98.2	2.45	3.06	-0.61	0.159
Actinomycetota	*Corynebacterium*	100	94.6	2.27	2.93	-0.66	0.723
Bacillota	*Fenollaria*	100	85.7	2.09	0.36	1.73	**0.0012**
Bacillota	*Anaerococcus*	100	91.1	1.55	0.53	1.02	**0.0011**
Bacillota	*Negativicoccus*	100	87.5	1.23	1.57	-0.34	0.375
Actinomycetota	*Mobiluncus*	100	78.6	1.21	0.35	0.86	**0.0017**
Actinomycetota	*Actinotignum*	100	62.5	1.14	0.05	1.09	**<0.0001**
Bacillota	*Finegoldia*	100	92.9	1.03	5.09	-4.06	**0.0087**
Bacillota	*Dialister*	100	92.9	0.84	1.11	-0.27	0.353
Actinomycetota	*Schaalia*	97.3	87.5	0.15	0.09	0.06	**0.0136**
Actinomycetota	*Winkia*	85.3	60.7	0.15	0.09	0.06	0.741
Bacillota	*Aerococcus*	86.7	42.9	0.14	0.00	0.14	**<0.0001**
Bacillota	*Streptococcus*	85.3	71.4	0.14	0.03	0.11	**0.0365**
Bacillota	*Murdochiella*	94.7	57.1	0.10	0.02	0.08	0.0602
Fusobacteriota	*Fusobacterium*	77.3	46.4	0.05	0.00	0.05	**0.0023**
Pseudomonadota	*Acinetobacter*	0.0	80.4	0.00	0.01	-0.01	**<0.0001**
Bacillota	*Lactobacillus*	5.33	83.9	0.00	0.06	-0.06	**<0.0001**
Synergistota	*Jonquetella*	34.7	26.8	0.00	0.00	0.00	0.609

* Significant adj. p values (Wilcoxon with FDR adj.) are bolded

Supplemental Table 4.1. Spearman correlations in pediatric cohort of the 22 shared genera between pediatric and adult microbiomes

Genus	*Hoylesella*	*Peptoniphilus*	*Ezakiella*	*Varibaculum*	*Porphyromonas*	*Campylobacter*	*Corynebacterium*	*Prevotella*	*Staphylococcus*	*Fenollaria*	*Anaerococcus*	*Finegoldia*	*Mobiluncus*	*Actinotignum*	*Negativicoccus*	*Dialister*	*Streptococcus*	*Fusobacterium*	*Veillonella*	*Murdochiella*	*Propionimicrobium*	*Winkia*
Hoylesella	1.00	-0.15	-0.12	0.08	-0.12	0.01	-0.06	0.09	0.06	-0.02	-0.32	-0.11	0.10	-0.05	0.01	0.52	-0.05	0.22	-0.12	0.08	0.41	-0.22
Peptoniphilus	-0.15	1.00	0.30	-0.10	0.24	0.08	-0.23	0.14	-0.18	-0.03	0.28	-0.03	0.01	0.09	0.23	-0.11	-0.10	-0.14	-0.06	0.12	0.08	-0.04
Ezakiella	-0.12	0.30	1.00	0.27	0.22	0.31	-0.33	-0.02	-0.22	0.04	0.07	-0.24	-0.15	0.17	0.31	-0.13	-0.24	0.06	-0.01	0.00	0.21	-0.03
Varibaculum	0.08	-0.10	0.27	1.00	0.29	0.24	-0.15	-0.10	-0.05	0.14	-0.27	-0.08	-0.04	-0.07	0.27	-0.08	-0.03	0.00	-0.05	0.21	0.02	-0.10
Porphyromonas	-0.12	0.24	0.22	0.29	1.00	0.37	-0.36	-0.03	-0.25	0.20	0.06	-0.20	0.14	0.15	0.25	-0.06	0.08	-0.26	-0.21	0.16	-0.21	-0.19
Campylobacter	0.01	0.08	0.31	0.24	0.37	1.00	-0.30	-0.11	-0.29	0.20	-0.08	-0.13	0.01	0.18	0.27	-0.11	-0.03	-0.14	0.08	0.26	0.06	-0.09
Corynebacterium	-0.06	-0.23	-0.33	-0.15	-0.36	-0.30	1.00	-0.10	0.39	-0.05	0.15	0.31	0.18	-0.15	-0.24	-0.05	0.00	0.10	0.16	-0.15	0.03	0.24
Prevotella	0.09	0.14	-0.02	-0.10	-0.03	-0.11	-0.10	1.00	-0.14	-0.09	-0.08	-0.02	0.35	-0.03	0.21	0.39	-0.21	0.16	-0.11	0.14	-0.01	-0.09
Staphylococcus	0.06	-0.18	-0.22	-0.05	-0.25	-0.29	0.39	-0.14	1.00	-0.01	0.22	0.36	-0.03	-0.07	-0.20	-0.01	0.15	0.25	-0.01	0.07	0.13	0.28
Fenollaria	-0.02	-0.03	0.04	0.14	0.20	0.20	-0.05	-0.09	-0.01	1.00	-0.05	-0.28	0.01	-0.02	0.07	-0.05	0.07	0.12	-0.04	0.23	-0.07	-0.14
Anaerococcus	-0.32	0.28	0.07	-0.27	0.06	-0.08	0.15	-0.08	0.22	-0.05	1.00	0.39	-0.28	0.00	0.03	-0.25	0.10	0.06	-0.06	-0.03	-0.13	0.18
Finegoldia	-0.11	-0.03	-0.24	-0.08	-0.20	-0.13	0.31	-0.02	0.36	-0.28	0.39	1.00	0.06	-0.06	0.00	-0.03	0.29	0.03	-0.08	-0.02	-0.18	0.26
Mobiluncus	0.10	0.01	-0.15	-0.04	0.14	0.01	0.18	0.35	-0.03	0.01	-0.28	0.06	1.00	-0.10	0.15	0.22	-0.03	-0.05	0.01	0.11	0.03	-0.16
Actinotignum	-0.05	0.09	0.17	-0.07	0.15	0.18	-0.15	-0.03	-0.07	-0.02	0.00	-0.06	-0.10	1.00	0.18	-0.16	-0.04	0.12	-0.19	-0.02	0.05	-0.11
Negativicoccus	0.01	0.23	0.31	0.27	0.25	0.27	-0.24	0.21	-0.20	0.07	0.03	0.00	0.15	0.18	1.00	-0.38	-0.17	-0.17	-0.21	0.20	-0.02	-0.15
Dialister	0.52	-0.11	-0.13	-0.08	-0.06	-0.11	-0.05	0.39	-0.01	-0.05	-0.25	-0.03	0.22	-0.16	-0.38	1.00	-0.11	0.24	-0.13	0.06	0.18	-0.14
Streptococcus	-0.05	-0.10	-0.24	-0.03	0.08	-0.03	0.00	-0.21	0.15	0.07	0.10	0.29	-0.03	-0.04	-0.17	-0.11	1.00	0.01	0.12	0.04	-0.15	0.34
Fusobacterium	0.22	-0.14	0.06	0.00	-0.26	-0.14	0.10	0.16	0.25	0.12	0.06	0.03	-0.05	0.12	-0.17	0.24	0.01	1.00	-0.17	-0.03	0.32	-0.12
Veillonella	-0.12	-0.06	-0.01	-0.05	-0.21	0.08	0.16	-0.11	-0.01	-0.04	-0.06	-0.08	0.01	-0.19	-0.21	-0.13	0.12	-0.17	1.00	-0.07	0.14	0.41
Murdochiella	0.08	0.12	0.00	0.21	0.16	0.26	-0.15	0.14	0.07	0.23	-0.03	-0.02	0.11	-0.02	0.20	0.06	0.04	-0.03	-0.07	1.00	-0.09	0.04
Propionimicrobium	0.41	0.08	0.21	0.02	-0.21	0.06	0.03	-0.01	0.13	-0.07	-0.13	-0.18	0.03	0.05	-0.02	0.18	-0.15	0.32	0.14	-0.09	1.00	-0.05
Winkia	-0.22	-0.04	-0.03	-0.10	-0.19	-0.09	0.24	-0.09	0.28	-0.14	0.18	0.26	-0.16	-0.11	-0.15	-0.14	0.34	-0.12	0.41	0.04	-0.05	1.00

Supplemental Table 4.2. FDR-adj. p values of Spearman correlations in pediatric cohort of the 22 shared genera between pediatric and adult microbiomes

Genus	*Hoylesella*	*Peptoniphilus*	*Ezakiella*	*Varibaculum*	*Porphyromonas*	*Campylobacter*	*Corynebacterium*	*Prevotella*	*Staphylococcus*	*Fenollaria*	*Anaerococcus*	*Finegoldia*	*Mobiluncus*	*Actinotignum*	*Negativicoccus*	*Dialister*	*Streptococcus*	*Fusobacterium*	*Veillonella*	*Murdochiella*	*Propionimicrobium*	*Winkia*
Hoylesella	0.00	0.25	0.57	0.83	0.47	0.81	0.86	0.39	0.97	0.94	0.02	0.37	0.46	0.71	0.62	0.00	0.43	0.15	0.50	0.94	0.03	0.14
Peptoniphilus	0.25	0.00	0.06	0.90	0.13	0.38	0.09	0.64	0.10	0.94	0.31	0.55	0.83	0.45	0.09	0.36	0.37	0.23	0.57	0.70	0.85	0.57
Ezakiella	0.57	0.06	0.00	0.14	0.09	0.03	0.01	0.89	0.03	0.68	0.95	0.05	0.34	0.20	0.04	0.32	0.11	0.67	0.71	0.89	0.60	0.34
Varibaculum	0.83	0.90	0.14	0.00	0.08	0.06	0.12	0.64	0.24	0.26	0.14	0.20	0.89	0.94	0.11	0.64	0.59	0.60	0.59	0.23	0.94	0.25
Porphyromonas	0.47	0.13	0.09	0.08	0.00	0.01	0.00	0.94	0.01	0.20	0.87	0.14	0.89	0.32	0.03	0.42	0.83	0.07	0.23	0.23	0.20	0.14
Campylobacter	0.81	0.38	0.03	0.06	0.01	0.00	0.00	0.64	0.01	0.22	0.45	0.07	0.86	0.26	0.04	0.37	0.56	0.16	0.89	0.20	0.88	0.25
Corynebacterium	0.86	0.09	0.01	0.12	0.00	0.00	0.00	0.59	0.00	0.37	0.45	0.03	0.81	0.20	0.03	0.95	0.49	0.54	0.30	0.20	0.99	0.09
Prevotella	0.39	0.64	0.89	0.64	0.94	0.64	0.59	0.00	0.37	0.63	0.38	0.66	0.04	0.86	0.59	0.05	0.12	0.64	0.35	0.67	0.94	0.23
Staphylococcus	0.97	0.10	0.03	0.24	0.01	0.01	0.00	0.37	0.00	0.49	0.26	0.02	0.63	0.36	0.04	0.95	0.24	0.23	0.76	0.55	0.89	0.09
Fenollaria	0.94	0.94	0.68	0.26	0.20	0.22	0.37	0.63	0.49	0.00	0.56	0.08	0.94	0.94	0.63	0.74	0.95	0.99	0.67	0.21	0.64	0.27
Anaerococcus	0.02	0.31	0.95	0.14	0.87	0.45	0.45	0.38	0.26	0.56	0.00	0.04	0.09	0.95	0.89	0.09	0.43	0.89	0.94	0.56	0.25	0.20
Finegoldia	0.37	0.55	0.05	0.20	0.14	0.07	0.03	0.66	0.02	0.08	0.04	0.00	0.88	0.47	0.37	0.68	0.09	0.97	0.95	0.49	0.24	0.07
Mobiluncus	0.46	0.83	0.34	0.89	0.89	0.86	0.81	0.04	0.63	0.94	0.09	0.88	0.00	0.49	0.80	0.20	0.55	0.78	0.80	0.73	0.94	0.27
Actinotignum	0.71	0.45	0.20	0.94	0.32	0.26	0.20	0.86	0.36	0.94	0.95	0.47	0.49	0.00	0.23	0.37	0.57	0.91	0.23	0.94	0.95	0.32
Negativicoccus	0.62	0.09	0.04	0.11	0.03	0.04	0.03	0.59	0.04	0.63	0.89	0.37	0.80	0.23	0.00	0.13	0.21	0.19	0.20	0.25	0.60	0.16
Dialister	0.00	0.36	0.32	0.64	0.42	0.37	0.95	0.05	0.95	0.74	0.09	0.68	0.20	0.37	0.13	0.00	0.49	0.14	0.59	0.97	0.23	0.32
Streptococcus	0.43	0.37	0.11	0.59	0.83	0.56	0.49	0.12	0.24	0.95	0.43	0.09	0.55	0.57	0.21	0.49	0.00	0.74	0.44	0.89	0.20	0.04
Fusobacterium	0.15	0.23	0.67	0.60	0.07	0.16	0.54	0.64	0.23	0.99	0.89	0.97	0.78	0.91	0.19	0.14	0.74	0.00	0.43	0.49	0.09	0.56
Veillonella	0.50	0.57	0.71	0.59	0.23	0.89	0.30	0.35	0.76	0.67	0.94	0.95	0.80	0.23	0.20	0.59	0.44	0.43	0.00	0.49	0.78	0.02
Murdochiella	0.94	0.70	0.89	0.23	0.23	0.20	0.20	0.67	0.55	0.21	0.56	0.49	0.73	0.94	0.25	0.97	0.89	0.49	0.49	0.00	0.38	0.57
Propionimicrobium	0.03	0.85	0.60	0.94	0.20	0.88	0.99	0.94	0.89	0.64	0.25	0.24	0.94	0.95	0.60	0.23	0.20	0.09	0.78	0.38	0.00	0.52
Winkia	0.14	0.57	0.34	0.25	0.14	0.25	0.09	0.23	0.09	0.27	0.20	0.07	0.27	0.32	0.16	0.32	0.04	0.56	0.02	0.57	0.52	0.00

Supplemental Table 4.3. Spearman correlations in adult cohort of the 22 shared genera between pediatric and adult microbiomes

Genus	*Hoylesella*	*Peptoniphilus*	*Ezakiella*	*Varibaculum*	*Porphyromonas*	*Campylobacter*	*Corynebacterium*	*Prevotella*	*Staphylococcus*	*Fenollaria*	*Anaerococcus*	*Finegoldia*	*Mobiluncus*	*Actinotignum*	*Negativicoccus*	*Dialister*	*Streptococcus*	*Fusobacterium*	*Veillonella*	*Murdochiella*	*Propionimicrobium*	*Winkia*
Hoylesella	1.00	0.12	0.38	0.47	0.19	0.49	-0.19	-0.07	-0.53	0.23	-0.48	-0.35	0.29	0.50	0.31	0.29	-0.21	0.22	-0.24	0.29	0.43	-0.40
Peptoniphilus	0.12	1.00	0.06	0.04	0.31	-0.01	-0.09	0.12	-0.03	-0.14	0.16	0.15	-0.03	-0.13	0.40	-0.06	0.08	0.22	0.17	0.14	0.25	0.20
Ezakiella	0.38	0.06	1.00	0.50	0.52	0.48	-0.38	0.02	-0.31	0.33	-0.34	-0.66	0.48	0.34	0.14	0.24	-0.40	0.21	-0.11	0.48	0.55	-0.39
Varibaculum	0.47	0.04	0.50	1.00	0.30	0.58	-0.23	0.08	-0.50	0.33	-0.32	-0.46	0.26	0.41	0.05	0.34	-0.27	0.15	-0.04	0.39	0.50	-0.30
Porphyromonas	0.19	0.31	0.52	0.30	1.00	0.27	-0.52	0.12	-0.30	0.37	-0.04	-0.49	0.27	0.11	0.33	0.33	-0.26	0.07	-0.23	0.61	0.42	-0.40
Campylobacter	0.49	-0.01	0.48	0.58	0.27	1.00	-0.33	0.07	-0.52	0.21	-0.46	-0.52	0.33	0.63	0.19	0.27	-0.28	0.35	-0.24	0.22	0.64	-0.39
Corynebacterium	-0.19	-0.09	-0.38	-0.23	-0.52	-0.33	1.00	-0.27	0.45	-0.06	0.42	0.54	-0.30	-0.20	-0.07	-0.37	0.14	-0.14	0.27	-0.36	-0.43	0.48
Prevotella	-0.07	0.12	0.02	0.08	0.12	0.07	-0.27	1.00	-0.13	0.21	-0.01	-0.09	0.02	0.07	-0.27	0.37	0.26	0.08	0.22	0.25	0.02	-0.08
Staphylococcus	-0.53	-0.03	-0.31	-0.50	-0.30	-0.52	0.45	-0.13	1.00	-0.12	0.49	0.37	-0.32	-0.58	-0.08	-0.53	0.17	-0.18	0.33	-0.31	-0.46	0.35
Fenollaria	0.23	-0.14	0.33	0.33	0.37	0.21	-0.06	0.21	-0.12	1.00	-0.10	-0.33	0.10	0.19	-0.08	0.23	-0.13	-0.02	-0.22	0.39	0.12	-0.26
Anaerococcus	-0.48	0.16	-0.34	-0.32	-0.04	-0.46	0.42	-0.01	0.49	-0.10	1.00	0.53	-0.58	-0.47	0.06	-0.25	0.34	-0.06	0.36	-0.19	-0.38	0.48
Finegoldia	-0.35	0.15	-0.66	-0.46	-0.49	-0.52	0.54	-0.09	0.37	-0.33	0.53	1.00	-0.52	-0.47	0.00	-0.38	0.51	-0.19	0.27	-0.44	-0.54	0.75
Mobiluncus	0.29	-0.03	0.48	0.26	0.27	0.33	-0.30	0.02	-0.32	0.10	-0.58	-0.52	1.00	0.26	0.04	0.35	-0.35	0.05	-0.17	0.38	0.33	-0.43
Actinotignum	0.50	-0.13	0.34	0.41	0.11	0.63	-0.20	0.07	-0.58	0.19	-0.47	-0.47	0.26	1.00	-0.02	0.24	-0.10	0.38	-0.25	0.09	0.50	-0.30
Negativicoccus	0.31	0.40	0.14	0.05	0.33	0.19	-0.07	-0.27	-0.08	-0.08	0.06	0.00	0.04	-0.02	1.00	-0.22	-0.30	0.04	-0.15	0.07	0.08	-0.05
Dialister	0.29	-0.06	0.24	0.34	0.33	0.27	-0.37	0.37	-0.53	0.23	-0.25	-0.38	0.35	0.24	-0.22	1.00	0.13	0.10	-0.13	0.49	0.33	-0.39
Streptococcus	-0.21	0.08	-0.40	-0.27	-0.26	-0.28	0.14	0.26	0.17	-0.13	0.34	0.51	-0.35	-0.10	-0.30	0.13	1.00	0.07	0.36	-0.14	-0.15	0.33
Fusobacterium	0.22	0.22	0.21	0.15	0.07	0.35	-0.14	0.08	-0.18	-0.02	-0.06	-0.19	0.05	0.38	0.04	0.10	0.07	1.00	0.07	0.05	0.28	-0.12
Veillonella	-0.24	0.17	-0.11	-0.04	-0.23	-0.24	0.27	0.22	0.33	-0.22	0.36	0.27	-0.17	-0.25	-0.15	-0.13	0.36	0.07	1.00	-0.05	-0.20	0.33
Murdochiella	0.29	0.14	0.48	0.39	0.61	0.22	-0.36	0.25	-0.31	0.39	-0.19	-0.44	0.38	0.09	0.07	0.49	-0.14	0.05	-0.05	1.00	0.32	-0.36
Propionimicrobium	0.43	0.25	0.55	0.50	0.42	0.64	-0.43	0.02	-0.46	0.12	-0.38	-0.54	0.33	0.50	0.08	0.33	-0.15	0.28	-0.20	0.32	1.00	-0.38
Winkia	-0.40	0.20	-0.39	-0.30	-0.40	-0.39	0.48	-0.08	0.35	-0.26	0.48	0.75	-0.43	-0.30	-0.05	-0.39	0.33	-0.12	0.33	-0.36	-0.38	1.00

Supplemental Table 4.4. FDR-adj. p values of Spearman correlations in adult cohort of the 22 shared genera between pediatric and adult microbiomes

Genus	*Hoylesella*	*Peptoniphilus*	*Ezakiella*	*Varibaculum*	*Porphyromonas*	*Campylobacter*	*Corynebacterium*	*Prevotella*	*Staphylococcus*	*Fenollaria*	*Anaerococcus*	*Finegoldia*	*Mobiluncus*	*Actinotignum*	*Negativicoccus*	*Dialister*	*Streptococcus*	*Fusobacterium*	*Veillonella*	*Murdochiella*	*Propionimic*	*Wi*
Hoylesella	0.00	0.87	0.00	0.00	0.00	0.00	0.00	0.96	0.00	0.01	0.00	0.00	0.00	0.00	0.10	0.00	0.00	0.02	0.00	0.00	0.00	0.00
Peptoniphilus	0.87	0.00	0.81	0.62	0.43	0.63	0.68	0.87	0.85	0.13	0.44	0.55	0.57	0.34	0.02	0.44	0.88	0.60	0.60	0.98	0.82	0.50
Ezakiella	0.00	0.81	0.00	0.00	0.00	0.00	0.00	0.62	0.00	0.00	0.00	7.029	0.00	0.00	0.24	0.00	0.00	0.04	0.00	0.00	0.00	0.00
Varibaculum	0.00	0.62	0.00	0.00	0.00	0.00	0.00	0.49	0.00	0.00	0.00	0.00	0.00	0.00	0.46	0.00	0.00	0.04	0.00	0.00	0.00	0.00
Porphyromonas	0.00	0.43	0.00	0.00	0.00	0.00	0.00	0.38	0.00	0.00	0.00	0.00	0.00	0.02	0.07	0.00	0.00	0.20	0.00	0.00	0.00	0.00
Campylobacter	0.00	0.63	0.00	0.00	0.00	0.00	0.00	0.63	0.00	0.01	0.00	0.00	0.00	0.00	0.28	0.00	0.00	0.00	0.00	0.00	0.00	0.00
Corynebacterium	0.00	0.68	0.00	0.00	0.00	0.00	0.00	0.07	0.00	0.01	0.00	0.00	0.00	0.00	0.36	0.00	0.01	0.02	0.00	0.00	0.00	0.00
Prevotella	0.96	0.87	0.62	0.49	0.38	0.63	0.07	0.00	0.30	0.21	0.53	0.37	0.57	0.59	0.03	0.01	0.41	0.63	0.74	0.10	0.57	0.32
Staphylococcus	0.00	0.85	0.00	0.00	0.00	0.00	0.00	0.30	0.00	0.01	0.00	0.00	0.00	0.00	0.41	0.00	0.00	0.01	0.00	0.00	0.00	0.00
Fenollaria	0.01	0.13	0.00	0.00	0.00	0.01	0.01	0.21	0.01	0.00	0.01	0.00	0.01	0.01	0.94	0.00	0.01	0.60	0.00	0.00	0.01	0.00
Anaerococcus	0.00	0.44	0.00	0.00	0.00	0.00	0.00	0.53	0.00	0.01	0.00	0.00	0.00	0.00	0.53	0.00	0.00	0.03	0.00	0.00	0.00	0.00
Finegoldia	0.00	0.55	0.00	0.00	0.00	0.00	0.00	0.37	0.00	0.00	0.00	0.00	0.00	0.00	0.43	0.00	0.00	0.02	0.00	0.00	0.00	0.00
Mobiluncus	0.00	0.57	0.00	0.00	0.00	0.00	0.00	0.57	0.00	0.01	0.00	0.00	0.00	0.00	0.44	0.00	0.00	0.11	0.00	0.00	0.00	0.00
Actinotignum	0.00	0.34	0.00	0.00	0.02	0.00	0.00	0.59	0.00	0.01	0.00	0.00	0.00	0.00	0.67	0.00	0.01	0.00	0.00	0.01	0.00	0.00
Negativicoccus	0.10	0.02	0.24	0.46	0.07	0.28	0.36	0.03	0.41	0.94	0.53	0.43	0.44	0.67	0.00	0.57	0.01	0.66	0.09	0.53	0.29	0.41
Dialister	0.00	0.44	0.00	0.00	0.00	0.00	0.00	0.01	0.00	0.00	0.00	0.00	0.00	0.00	0.57	0.00	0.11	0.12	0.01	0.00	0.00	0.00
Streptococcus	0.00	0.88	0.00	0.00	0.00	0.00	0.01	0.41	0.00	0.01	0.00	0.00	0.00	0.01	0.01	0.11	0.00	0.28	0.00	0.00	0.00	0.00
Fusobacterium	0.02	0.60	0.04	0.04	0.20	0.00	0.02	0.63	0.01	0.60	0.03	0.02	0.11	0.00	0.66	0.12	0.28	0.00	0.21	0.28	0.01	0.03
Veillonella	0.00	0.60	0.00	0.00	0.00	0.00	0.00	0.74	0.00	0.00	0.00	0.00	0.00	0.00	0.09	0.01	0.00	0.21	0.00	0.00	0.00	0.00
Murdochiella	0.00	0.98	0.00	0.00	0.00	0.00	0.00	0.10	0.00	0.00	0.00	0.00	0.00	0.01	0.53	0.00	0.00	0.28	0.00	0.00	0.00	0.00
Propionimicrobium	0.00	0.82	0.00	0.00	0.00	0.00	0.00	0.57	0.00	0.01	0.00	0.00	0.00	0.00	0.29	0.00	0.00	0.01	0.00	0.00	0.00	0.00
Winkia	0.00	0.50	0.00	0.00	0.00	0.00	0.00	0.32	0.00	0.00	0.00	7.611	0.00	0.00	0.41	0.00	0.00	0.03	0.00	0.00	0.00	0.00

Supplemental Table 5. Spearman's correlations values and difference between adults and pediatric of the 22 common genera among the top 30 most abundant genera.

Taxa Pair	ρ ped	ρ adult	Δρ = ρ adult - ρ ped
Propionimicrobium | Porphyromonas	-0.212	0.423	0.635
Mobiluncus | Ezakiella	-0.149	0.475	0.624
Staphylococcus | Hoylesella	0.065	-0.528	-0.593
Propionimicrobium | Staphylococcus	0.129	-0.462	-0.591
Propionimicrobium | Campylobacter	0.061	0.636	0.574
Mobiluncus | Finegoldia	0.056	-0.515	-0.572
Actinotignum | Hoylesella	-0.053	0.505	0.557
Dialister | Staphylococcus	-0.011	-0.533	-0.522
Actinotignum | Staphylococcus	-0.068	-0.575	-0.508
Ezakiella | Hoylesella	-0.124	0.375	0.499
Fusobacterium | Campylobacter	-0.144	0.353	0.497
Winkia | Finegoldia	0.260	0.755	0.495
Campylobacter | Hoylesella	0.009	0.491	0.482
Negativicoccus | Prevotella	0.209	-0.271	-0.480
Actinotignum | Varibaculum	-0.067	0.412	0.479
Propionimicrobium | Varibaculum	0.017	0.496	0.479
Murdochiella | Ezakiella	-0.001	0.476	0.478
Mobiluncus | Corynebacterium	0.176	-0.300	-0.476
Streptococcus | Prevotella	-0.213	0.260	0.472
Actinotignum | Anaerococcus	-0.002	-0.472	-0.471
Propionimicrobium | Corynebacterium	0.027	-0.432	-0.459
Murdochiella | Porphyromonas	0.157	0.609	0.452
Actinotignum | Campylobacter	0.179	0.630	0.451
Propionimicrobium | Actinotignum	0.052	0.501	0.449
Staphylococcus | Varibaculum	-0.053	-0.500	-0.447
Murdochiella | Dialister	0.056	0.490	0.434
Fusobacterium | Staphylococcus	0.247	-0.179	-0.426
Finegoldia | Ezakiella	-0.239	-0.663	-0.424
Dialister | Varibaculum	-0.079	0.345	0.424
Veillonella | Anaerococcus	-0.064	0.357	0.421
Murdochiella | Finegoldia	-0.024	-0.441	-0.416
Propionimicrobium | Murdochiella	-0.089	0.324	0.413
Anaerococcus | Ezakiella	0.074	-0.336	-0.410
Dialister | Actinotignum	-0.163	0.240	0.403
Actinotignum | Finegoldia	-0.065	-0.467	-0.402

Winkia | Murdochiella	0.038	-0.358	-0.397
Dialister | Porphyromonas	-0.064	0.327	0.390
Finegoldia | Campylobacter	-0.133	-0.521	-0.388
Varibaculum | Hoylesella	0.084	0.469	0.384
Finegoldia | Varibaculum	-0.076	-0.455	-0.379
Anaerococcus | Campylobacter	-0.082	-0.458	-0.376
Dialister | Campylobacter	-0.107	0.267	0.374
Murdochiella | Staphylococcus	0.067	-0.306	-0.373
Dialister | Ezakiella	-0.128	0.244	0.372
Winkia | Ezakiella	-0.028	-0.395	-0.367
Actinotignum | Mobiluncus	-0.097	0.263	0.360
Fusobacterium | Peptoniphilus	-0.141	0.216	0.357
Propionimicrobium | Finegoldia	-0.181	-0.538	-0.357
Dialister | Finegoldia	-0.029	-0.382	-0.354
Veillonella | Finegoldia	-0.082	0.269	0.351
Propionimicrobium | Veillonella	0.143	-0.202	-0.344
Streptococcus | Porphyromonas	0.084	-0.258	-0.342
Mobiluncus | Prevotella	0.354	0.016	-0.338
Campylobacter | Varibaculum	0.245	0.583	0.338
Veillonella | Staphylococcus	-0.009	0.328	0.337
Propionimicrobium | Ezakiella	0.213	0.549	0.335
Winkia | Propionimicrobium	-0.052	-0.384	-0.332
Fusobacterium | Porphyromonas	-0.259	0.070	0.329
Veillonella | Prevotella	-0.108	0.217	0.324
Mobiluncus | Campylobacter	0.010	0.334	0.324
Dialister | Corynebacterium	-0.045	-0.368	-0.323
Veillonella | Campylobacter	0.079	-0.239	-0.318
Streptococcus | Mobiluncus	-0.034	-0.349	-0.315
Porphyromonas | Hoylesella	-0.121	0.190	0.312
Mobiluncus | Varibaculum	-0.044	0.264	0.308
Negativicoccus | Hoylesella	0.006	0.310	0.304
Winkia | Anaerococcus	0.177	0.480	0.303
Winkia | Campylobacter	-0.089	-0.389	-0.300
Porphyromonas | Ezakiella	0.220	0.519	0.299
Mobiluncus | Anaerococcus	-0.280	-0.578	-0.298
Fenollaria | Ezakiella	0.036	0.333	0.298
Propionimicrobium | Mobiluncus	0.028	0.325	0.297
Finegoldia | Porphyromonas	-0.196	-0.492	-0.295

Mobiluncus | *Staphylococcus*	-0.029	-0.323	-0.295
Fenollaria | *Prevotella*	-0.085	0.208	0.294
Dialister | *Fenollaria*	-0.052	0.231	0.283
Anaerococcus | *Corynebacterium*	0.145	0.420	0.274
Murdochiella | *Mobiluncus*	0.113	0.384	0.272
Winkia | *Mobiluncus*	-0.160	-0.432	-0.271
Peptoniphilus | *Hoylesella*	-0.152	0.118	0.271
Anaerococcus | *Staphylococcus*	0.221	0.488	0.267
Propionimicrobium | *Anaerococcus*	-0.126	-0.385	-0.259
Fusobacterium | *Actinotignum*	0.125	0.377	0.252
Streptococcus | *Campylobacter*	-0.027	-0.278	-0.251
Winkia | *Dialister*	-0.139	-0.387	-0.248
Veillonella | *Streptococcus*	0.117	0.365	0.248
Fenollaria | *Hoylesella*	-0.016	0.231	0.247
Veillonella | *Fusobacterium*	-0.170	0.072	0.242
Winkia | *Peptoniphilus*	-0.036	0.205	0.240
Winkia | *Corynebacterium*	0.240	0.480	0.240
Streptococcus | *Anaerococcus*	0.101	0.340	0.239
Finegoldia | *Hoylesella*	-0.114	-0.352	-0.238
Ezakiella | *Peptoniphilus*	0.295	0.057	-0.238
Staphylococcus | *Campylobacter*	-0.286	-0.521	-0.236
Streptococcus | *Varibaculum*	-0.033	-0.267	-0.234
Varibaculum | *Ezakiella*	0.267	0.500	0.233
Fusobacterium | *Corynebacterium*	0.098	-0.136	-0.233
Streptococcus | *Dialister*	-0.105	0.126	0.231
Finegoldia | *Corynebacterium*	0.314	0.545	0.230
Veillonella | *Peptoniphilus*	-0.062	0.167	0.228
Dialister | *Hoylesella*	0.518	0.289	-0.228
Negativicoccus | *Varibaculum*	0.270	0.053	-0.217
Fusobacterium | *Finegoldia*	0.027	-0.189	-0.216
Murdochiella | *Corynebacterium*	-0.146	-0.362	-0.215
Actinotignum | *Peptoniphilus*	0.087	-0.128	-0.215
Fusobacterium | *Negativicoccus*	-0.174	0.040	0.214
Streptococcus | *Finegoldia*	0.294	0.507	0.213
Actinotignum | *Fenollaria*	-0.025	0.186	0.211
Winkia | *Porphyromonas*	-0.189	-0.399	-0.210
Murdochiella | *Hoylesella*	0.083	0.288	0.205
Streptococcus | *Fenollaria*	0.071	-0.134	-0.204

Negativicoccus | Actinotignum	0.176	-0.023	-0.199
Mobiluncus | Hoylesella	0.097	0.291	0.194
Winkia | Varibaculum	-0.105	-0.298	-0.193
Fenollaria | Varibaculum	0.144	0.333	0.190
Winkia | Actinotignum	-0.113	-0.302	-0.190
Streptococcus | Peptoniphilus	-0.103	0.083	0.186
Propionimicrobium | Fenollaria	-0.067	0.116	0.183
Veillonella | Mobiluncus	0.013	-0.168	-0.182
Finegoldia | Peptoniphilus	-0.035	0.145	0.180
Veillonella | Fenollaria	-0.040	-0.220	-0.180
Campylobacter | Ezakiella	0.305	0.485	0.180
Murdochiella | Streptococcus	0.043	-0.136	-0.179
Prevotella | Varibaculum	-0.099	0.079	0.178
Murdochiella | Varibaculum	0.214	0.391	0.177
Prevotella | Campylobacter	-0.105	0.072	0.177
Winkia | Hoylesella	-0.221	-0.397	-0.176
Negativicoccus | Corynebacterium	-0.244	-0.072	0.172
Actinotignum | Ezakiella	0.173	0.344	0.172
Negativicoccus | Ezakiella	0.306	0.136	-0.170
Fenollaria | Porphyromonas	0.200	0.369	0.169
Murdochiella | Fenollaria	0.227	0.395	0.168
Propionimicrobium | Peptoniphilus	0.082	0.250	0.168
Corynebacterium | Porphyromonas	-0.357	-0.524	-0.167
Prevotella | Corynebacterium	-0.101	-0.268	-0.167
Streptococcus | Hoylesella	-0.050	-0.215	-0.165
Streptococcus | Ezakiella	-0.235	-0.399	-0.164
Dialister | Negativicoccus	-0.382	-0.219	0.164
Negativicoccus | Peptoniphilus	0.234	0.395	0.161
Prevotella | Hoylesella	0.095	-0.065	-0.160
Anaerococcus | Hoylesella	-0.325	-0.481	-0.156
Murdochiella | Anaerococcus	-0.031	-0.186	-0.155
Fusobacterium | Ezakiella	0.058	0.209	0.152
Staphylococcus | Peptoniphilus	-0.179	-0.028	0.151
Fusobacterium | Varibaculum	0.002	0.153	0.150
Prevotella | Porphyromonas	-0.031	0.118	0.149
Propionimicrobium | Dialister	0.184	0.327	0.143
Negativicoccus | Fenollaria	0.068	-0.075	-0.143
Varibaculum | Peptoniphilus	-0.099	0.044	0.143

Fusobacterium | Dialister	0.239	0.096	-0.143
Streptococcus | Corynebacterium	-0.003	0.139	0.142
Corynebacterium | Peptoniphilus	-0.231	-0.090	0.141
Finegoldia | Anaerococcus	0.388	0.530	0.141
Mobiluncus | Porphyromonas	0.138	0.274	0.136
Streptococcus | Negativicoccus	-0.169	-0.305	-0.136
Fusobacterium | Fenollaria	0.120	-0.015	-0.135
Corynebacterium | Hoylesella	-0.056	-0.186	-0.130
Murdochiella | Negativicoccus	0.199	0.071	-0.129
Winkia | Fenollaria	-0.139	-0.263	-0.124
Dialister | Mobiluncus	0.222	0.346	0.124
Negativicoccus | Staphylococcus	-0.205	-0.082	0.123
Fusobacterium | Anaerococcus	0.064	-0.057	-0.121
Anaerococcus | Peptoniphilus	0.281	0.163	-0.118
Fenollaria | Peptoniphilus	-0.025	-0.141	-0.116
Veillonella | Hoylesella	-0.125	-0.237	-0.113
Murdochiella | Prevotella	0.139	0.250	0.112
Murdochiella | Actinotignum	-0.020	0.091	0.111
Veillonella | Corynebacterium	0.159	0.266	0.107
Propionimicrobium | Negativicoccus	-0.023	0.084	0.107
Negativicoccus | Mobiluncus	0.151	0.045	-0.106
Veillonella | Ezakiella	-0.006	-0.112	-0.105
Fenollaria | Staphylococcus	-0.013	-0.117	-0.104
Fusobacterium | Mobiluncus	-0.050	0.051	0.101
Anaerococcus | Porphyromonas	0.060	-0.038	-0.099
Campylobacter | Porphyromonas	0.371	0.273	-0.099
Actinotignum | Prevotella	-0.032	0.066	0.098
Winkia | Negativicoccus	-0.147	-0.053	0.094
Campylobacter | Peptoniphilus	0.082	-0.009	-0.091
Staphylococcus | Ezakiella	-0.221	-0.312	-0.090
Negativicoccus | Campylobacter	0.272	0.185	-0.087
Mobiluncus | Fenollaria	0.012	0.095	0.083
Corynebacterium | Varibaculum	-0.146	-0.228	-0.081
Winkia | Veillonella	0.410	0.330	-0.081
Fusobacterium | Prevotella	0.159	0.078	-0.080
Murdochiella | Fusobacterium	-0.026	0.053	0.079
Negativicoccus | Porphyromonas	0.253	0.332	0.078
Winkia | Staphylococcus	0.275	0.352	0.077

Porphyromonas | Peptoniphilus	0.237	0.314	0.077
Anaerococcus | Prevotella	-0.083	-0.010	0.073
Finegoldia | Prevotella	-0.017	-0.086	-0.069
Veillonella | Negativicoccus	-0.215	-0.146	0.069
Fusobacterium | Streptococcus	0.006	0.071	0.065
Staphylococcus | Corynebacterium	0.389	0.449	0.061
Streptococcus | Actinotignum	-0.043	-0.104	-0.061
Veillonella | Actinotignum	-0.190	-0.249	-0.059
Anaerococcus | Fenollaria	-0.046	-0.102	-0.056
Finegoldia | Fenollaria	-0.282	-0.334	-0.052
Staphylococcus | Porphyromonas	-0.251	-0.302	-0.051
Corynebacterium | Ezakiella	-0.335	-0.383	-0.049
Dialister | Peptoniphilus	-0.108	-0.061	0.048
Actinotignum | Corynebacterium	-0.152	-0.200	-0.047
Anaerococcus | Varibaculum	-0.271	-0.318	-0.047
Prevotella | Ezakiella	-0.022	0.021	0.043
Mobiluncus | Peptoniphilus	0.014	-0.028	-0.042
Murdochiella | Campylobacter	0.256	0.215	-0.041
Actinotignum | Porphyromonas	0.145	0.108	-0.037
Propionimicrobium | Fusobacterium	0.319	0.282	-0.037
Corynebacterium | Campylobacter	-0.299	-0.334	-0.034
Negativicoccus | Anaerococcus	0.030	0.058	0.028
Prevotella | Peptoniphilus	0.143	0.117	-0.026
Propionimicrobium | Prevotella	-0.006	0.019	0.025
Murdochiella | Peptoniphilus	0.115	0.136	0.021
Streptococcus | Staphylococcus	0.154	0.173	0.020
Veillonella | Porphyromonas	-0.211	-0.230	-0.020
Winkia | Streptococcus	0.345	0.328	-0.017
Propionimicrobium | Hoylesella	0.411	0.427	0.016
Veillonella | Varibaculum	-0.052	-0.036	0.016
Dialister | Prevotella	0.390	0.374	-0.016
Murdochiella | Veillonella	-0.067	-0.053	0.015
Finegoldia | Staphylococcus	0.360	0.375	0.014
Fenollaria | Campylobacter	0.198	0.212	0.014
Staphylococcus | Prevotella	-0.142	-0.130	0.012
Fenollaria | Corynebacterium	-0.052	-0.063	-0.010
Winkia | Prevotella	-0.089	-0.079	0.010
Porphyromonas | Varibaculum	0.295	0.304	0.009

Winkia | *Fusobacterium*	-0.116	-0.125	-0.009
Veillonella | *Dialister*	-0.125	-0.134	-0.008
Negativicoccus | *Finegoldia*	-0.004	-0.001	0.003
Fusobacterium | *Hoylesella*	0.221	0.222	0.001
Propionimicrobium | *Streptococcus*	-0.151	-0.151	-0.001
Dialister | *Anaerococcus*	-0.246	-0.246	0.000

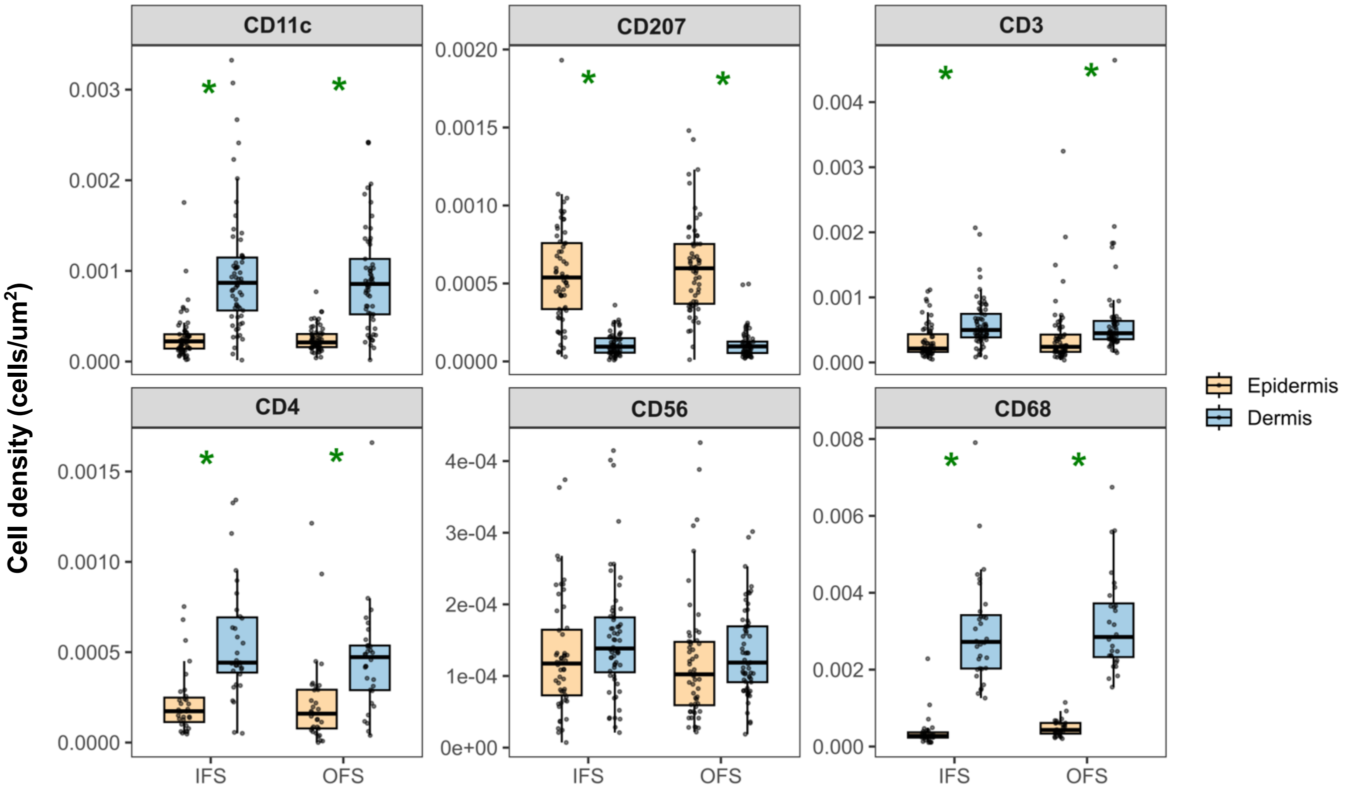

Supplemental Figure 1. Immune-cell densities in pediatric foreskin compartments. Box-plots and points show the distribution of cell densities (cells mm^{-2}) for each immune marker—CD3, CD4, CD11c, CD56, CD68 and CD207—stratified by anatomical location (IFS = inner foreskin, OFS = outer foreskin) and tissue layer (epidermis vs dermis). Within each site the two boxes correspond to Epidermis (left, yellow fill) and Dermis (right, blue fill); black outlines mark box borders and all individual observations are over-plotted. Paired Wilcoxon tests comparing dermis and epidermis for each site are indicated above the boxes as green stars ("ns" omitted), with all adj $p < 6x10^{-5}$. The figure highlights the markedly higher dermal densities of $CD68^+$, $CD3^+$, $CD4^+$ and $CD11c^+$ cells, the rarity of $CD56^+$ and $CD207^+$ cells in the dermis, and the predominance of $CD207^+$ Langerhans cells in the epidermis.

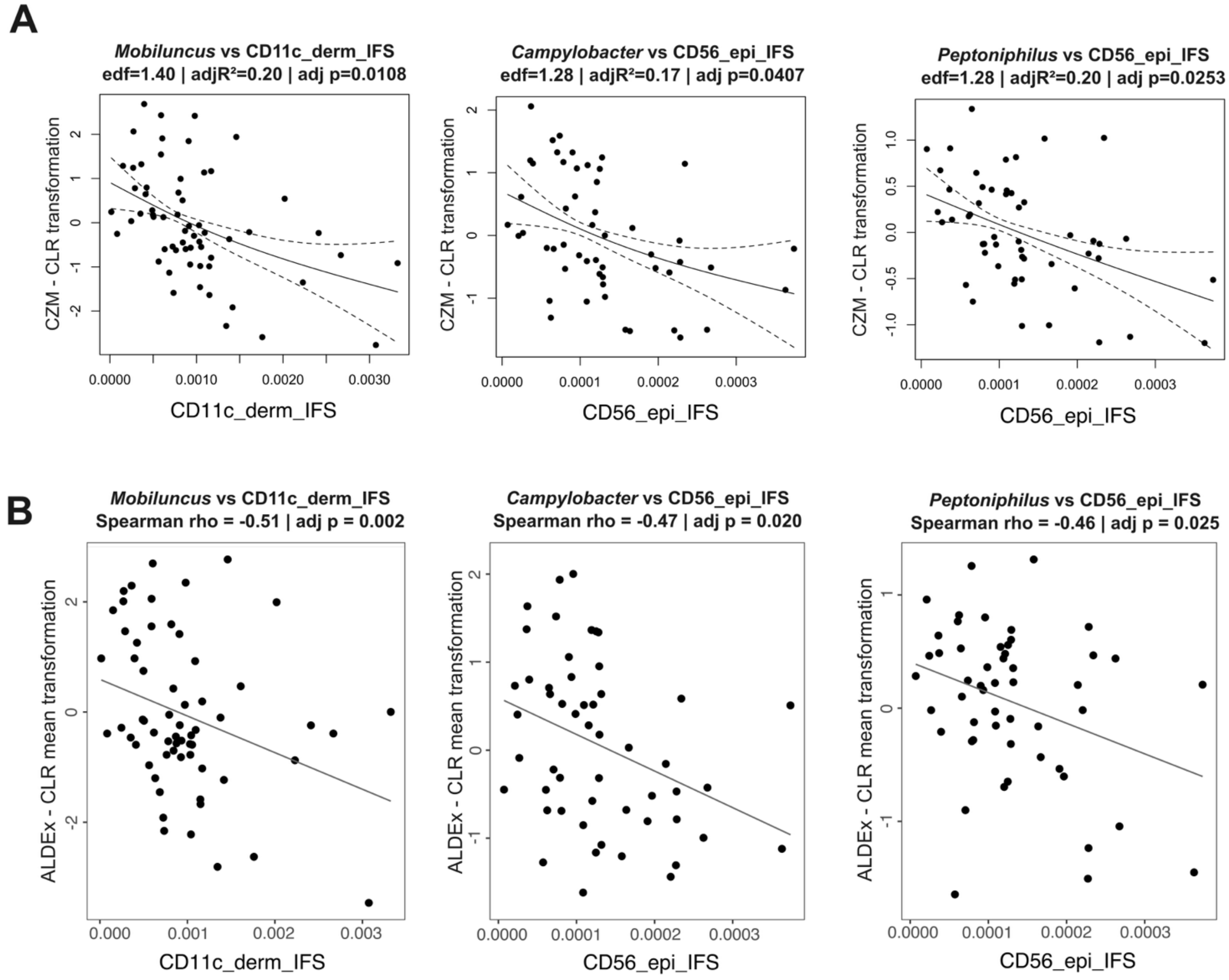

Supplemental Figure 2. Cross-validated associations between bacterial genera and immune-cell densities in the inner foreskin. (**A**) Generalized additive model (GAM) smooths for the three genus–marker pairs that met the FDR threshold (adj $p \leq 0.05$) in the CZM + CLR pipeline: *Mobiluncus* versus $CD11c^{+}$-cell density in the inner-dermal foreskin (CD11c_derm_IFS) and *Campylobacter* and *Peptoniphilus* versus $CD56^{+}$-cell density in the inner-epidermal foreskin (CD56_epi_IFS). Black curves are the fitted smooths, dashed lines their 95 % confidence intervals, and points the partial residuals; each panel title reports effective degrees of freedom (edf), adj R^2 and adj p. (**B**) Scatter-plots of the same genus–marker pairs based on ALDEx2 CLR mean values. Points represent individual participants; black lines are Spearman rank trends with 95 % confidence bands, and insets list the median ρ and FDR-adjusted q. In both analyses higher $CD11c^{+}$ density coincides with lower *Mobiluncus* abundance, while higher $CD56^{+}$ density corresponds to lower abundances of *Campylobacter* and *Peptoniphilus*. IFS = inner foreskin; epi = epidermis; derm = dermis.
